# CD38 marks the exhausted CD8^+^ tissue-resident memory T cells in hepatocellular carcinoma

**DOI:** 10.3389/fimmu.2023.1182016

**Published:** 2023-06-12

**Authors:** Marie J. Y. Reolo, Masayuki Otsuka, Justine Jia Wen Seow, Joycelyn Lee, Yun Hua Lee, Phuong H. D. Nguyen, Chun Jye Lim, Martin Wasser, Camillus Chua, Tony K. H. Lim, Wei Qiang Leow, Alexander Chung, Brian K. P. Goh, Pierce K. H. Chow, Ramanuj DasGupta, Joe Poh Sheng Yeong, Valerie Chew

**Affiliations:** ^1^ Translational Immunology Institute (TII), SingHealth-DukeNUS Academic Medical Centre, Singapore, Singapore; ^2^ Genome Institute of Singapore (GIS), Agency for Science, Technology and Research (A*STAR), Singapore, Singapore; ^3^ Division of Medical Oncology, National Cancer Centre Singapore, Singapore, Singapore; ^4^ Department of Anatomical Pathology, Singapore General Hospital, Singapore, Singapore; ^5^ Department of Hepatopancreatobiliary and Transplant Surgery, Division of Surgery and Surgical Oncology, Singapore General Hospital and National Cancer Centre Singapore, Singapore, Singapore; ^6^ SingHealth-DukeNUS Academic Surgery Program, Duke-NUS Graduate Medical School, Singapore, Singapore; ^7^ Division of Medical Science, National Cancer Center, Singapore, Singapore

**Keywords:** CD38, PD-1, T cell exhaustion, immunotherapy, HCC, immune checkpoint, tissue resident T cells

## Abstract

**Introduction:**

Despite recent advances in immunotherapy for hepatocellular carcinoma (HCC), the overall modest response rate underscores the need for a better understanding of the tumor microenvironment (TME) of HCC. We have previously shown that CD38 is widely expressed on tumor-infiltrating leukocytes (TILs), predominantly on CD3^+^ T cells and monocytes. However, its specific role in the HCC TME remains unclear.

**Methods:**

In this current study, we used cytometry time-of-flight (CyTOF), bulk RNA sequencing on sorted T cells, and single-cell RNA (scRNA) sequencing to interrogate expression of CD38 and its correlation with T cell exhaustion in HCC samples. We also employed multiplex immunohistochemistry (mIHC) for validating our findings.

**Results:**

From CyTOF analysis, we compared the immune composition of CD38-expressing leukocytes in TILs, non-tumor tissue-infiltrating leukocytes (NIL), and peripheral blood mononuclear cells (PBMC). We identified CD8^+^ T cells as the dominant CD38-expressing TILs and found that CD38 expression was significantly higher in CD8^+^ T_RM_ in TILs than in NILs. Furthermore, through transcriptomic analysis on sorted CD8^+^ T_RM_ from HCC tumors, we observed a higher expression of CD38 along with T cell exhaustion genes, including PDCD1 and CTLA4, compared to the circulating memory CD8 T cells from PBMC. This was validated by scRNA sequencing that revealed co-expression of CD38 with PDCD1, CTLA4, and ITGAE (CD103) in T cells from HCC tumors. The protein co-expression of CD38 and PD-1 on CD8^+^ T cells was further demonstrated by mIHC on HCC FFPE tissues, marking CD38 as a T cell co-exhaustion marker in HCC. Lastly, the higher proportions of CD38^+^PD-1^+^ CD8^+^ T cells and CD38^+^PD-1^+^ T_RM_ were significantly associated with the higher histopathological grades of HCC, indicating its role in the aggressiveness of the disease.

**Conclusion:**

Taken together, the concurrent expression of CD38 with exhaustion markers on CD8^+^ T_RM_ underpins its role as a key marker of T cell exhaustion and a potential therapeutic target for restoring cytotoxic T cell function in HCC.

## 1 Introduction

Hepatocellular carcinoma (HCC) accounts for at least 75% of primary liver cancer and is the third leading cause of cancer death globally (1). Inhibitors against programmed cell death protein (PD)-1 and its ligand PD-L1 or collectively known as Immune checkpoint blockades (ICBs), are the most recent therapeutic options for many advanced solid malignancies, including HCC (2, 3). However, the objective response rate (ORR) to ICB in patients with HCC remains modest at 10-20% for monotherapy (4, 5) or less than 30% for combination immunotherapy (6). The variation in clinical outcomes after immunotherapy underscores the need to better understand the immune landscape of HCC and to discover new biomarkers or therapeutic targets to improve clinical outcomes for patients with HCC.

CD38 is a multifunctional type II transmembrane glycoprotein with enzymatic functions that are involved in immune cell activation and regulation in homeostasis, inflammation and various diseases (7–9). Although initially thought to be expressed on T cells, CD38 is also expressed on other lymphoid and myeloid cell populations (7). CD38-expressing immune cells have been detected in the TME of numerous cancer types and are often associated with cancer progression (8, 10, 11). Therefore, there is a growing interest in CD38 as a novel therapeutic target for immune-based therapies, especially in solid tumors. In HCC, CD38 expression on myeloid cells has been associated with better HCC patient survival after surgery (12) and anti-PD1 therapy (13). Collectively, these studies suggest that CD38 has a promising potential as both an immunotherapeutic target and a biomarker for therapeutic response in HCC.

Our previous report has shown an association of CD38^+^ tumor-infiltrating leukocytes (TIL) with HCC prognosis and identified CD3^+^ T cells and monocytes as the dominant CD38-expressing immune populations among the TILs (14). While other studies have demonstrated the potential roles of CD38^+^ tumor-associated macrophages in the clinical outcome of HCC patients (12, 13), the role of CD38 in tumor-infiltrating T cells in the HCC TME remains to be further elucidated. In this study, we investigated the immune composition of CD38-expressing leukocytes in TILs, non-tumor tissue-infiltrating leukocytes (NILs), and peripheral blood mononuclear cells (PBMCs) using cytometry by time-of-flight (CyTOF), bulk and single-cell RNA sequencing as well as validation using multiplex immunohistochemistry (mIHC). We identified CD8^+^ T_RM_ cells as the predominant CD38-expressing immune cells in TILs, and their association with the T cell exhaustion signature in HCC TME and the higher histopathological tumor grades. Taken altogether, our findings show that CD38 is a marker of exhausted CD8^+^ T_RM_ cells in HCC that could be a potential therapeutic target in conjunction with other ICBs to restore cytotoxic T cell function.

## 2 Methods

### 2.1 Patient samples

Patient samples collection from the National University Hospital (NUH), National Cancer Center Singapore and Singapore General Hospital was approved by the NUH CIRB and SingHealth Central Institution Review Board (CIRB) (CIRB Ref: 2018/2112 and 2016/2626), respectively. Peripheral blood, tumor and adjacent non-tumor liver samples were collected from 17 HCC patients; each provided written informed consent, with demographics and clinical characteristics as described in **Table S1**. Depending on the tumor size, the tumors collected from each patient were dissected into two to five sectors (total tumor sectors= 60), separated by at least 1 cm to account for intratumoral heterogeneity (15). The adjacent non-tumor liver tissue, which is at least 2 cm away from the tumor, was also harvested. Each tissue sector was allocated for downstream analysis using Cytometry by Time-of-Flight (CyTOF), and bulk tissue RNA sequencing. A subset of them was subjected to single-cell RNA sequencing or mIHC, as described below. The peripheral blood mononuclear cells (PBMC) (n=17) were isolated from the blood using the Ficoll-Paque Plus (GE Healthcare) density gradient centrifugation. The tumor-infiltrating leukocytes (TIL) (from a total of n=60 tumor sectors) and non-tumor tissue-infiltrating leukocytes (NIL) (n=17) were isolated using enzymatic digestion with 500μg/mL collagenase IV (Thermo Fisher Scientific; Cat#: 17104019) and 50μg/mL DNase I (Roche, Indianapolis, IN; Cat#: 4716728001) for 30 min in 37°C. The cells were stored in liquid nitrogen with 10% DMSO in fetal bovine serum (FBS) until further analysis.

### 2.2 Cytometry by Time-of-Flight

TILs, NILs and PBMCs were thawed and rested for 1 h in RPMI medium with 10% FBS and 1% penicillin/streptomycin. The cells were stained as previously described (16) using a panel of 41 heavy-metal conjugated antibodies against surface and intracellular markers, including three anti-human CD45 barcode antibodies (**Table S2**). The data were obtained using Helios equipped with the CyTOF^®^ 6.7 system control software (Fluidigm).

The generated files were analyzed by FlowJo (v.10.2): live single-cells (cisplatin-negative and DNA-intercalator-positive) were debarcoded to each sample file based on their unique CD45 barcodes as previously described (17). The resulting data was down-sampled to 1x10^4^ cells/sample for subsequent analysis. Two-dimensional t-distributed (t-SNE) plots were generated to represent the expression of individual immune markers or various immune subsets. The data was further validated by manual gating using FlowJo (v.10.2).

### 2.3 Cell sorting and RNA sequencing

Tissue-resident memory CD8^+^ T cells (T_RM_) from TILs and matched circulating memory CD8^+^ T cells from PBMCs were sorted from seven HCC patients as previously described (16). Briefly, cells were stained with the fluorochrome-conjugated anti-human antibodies (**Table S3**) for 30 min, followed by DAPI staining (for live/dead cell stain). Then, using the FACS Aria II flow cytometer (BD Biosciences), the stained cells were sorted at an efficiency of 91%–100% into live T_RM_ (DAPI^-^CD45^+^CD3^+^CD8^+^CD45RO^+^CD103^+^) from TILs or circulating memory CD8^+^ T cells (DAPI^-^CD45^+^CD3^+^CD8^+^CD45RO^+^) from PBMCs for bulk RNA sequencing (**Figure S1A**).

For RNA sequencing, total RNAs from sorted CD8^+^ T_RM_ cells from TILs (n=7) and matched circulating memory CD8^+^ T cells from PBMCs (n=6; one sample was omitted due to poor RNA quality) were isolated using Picopure RNA-Isolation kit (Arcturus, Ambion) and cDNA was generated using the SMART-Seq® v4 UltraTM Low Input RNA Kit for Sequencing (Clontech, USA). With the Nextera XT DNA Library Prep Kit (Illumina, USA), indexed libraries were created and multiplexed for 2x 101 bp-sequencing. The raw reads were aligned to the Human Reference Genome hg19 via STAR (18) and the gene-level expected counts were calculated using RSEM (19). The samples with protein-coding genes of more than 0.5 counts were retained. Analyses on differentially expressed genes (DEGs) were performed using R packages DESeq2 and Limma (20) with the false discovery rate (FDR) adjusted for multiple testing using the Benjamini-Hochberg. Functional pathway analyses were performed using DAVID pathway analysis v.6.8, with adjusted p-value < 0.01.

### 2.4 Single-cell RNA sequencing

Single cells were isolated from tumor and adjacent non-tumor tissues from 14 patients with HCC by enzymatic digestion as described previously (21). Briefly, dead cells were removed using a dead cell removal kit (Miltenyi, Cat#: 130-090-101). CD45^+^ cells were enriched using CD45 MicroBeads (Miltenyi, Cat#: 130-045-801) before CD45^+^ and CD45^-^ cells were processed using the Chromium Single Cell 30 (v2 Chemistry) platform (10x Genomics, Pleasanton, CA). All data were then aggregated using cellranger aggr by normalizing all runs to the same sequencing depth. Downstream analysis was performed using Scanpy (version 1.8.1); All genes expressed by a minimum of 30 cells were considered, and cells with fewer than 200 genes and greater than 5% mitochondrial content were excluded from the analysis. A subset of T cells from the whole atlas was obtained. Leiden (scanpy.api.tl.leiden) with the resolution parameters set at 0.35 and 0.25 for NILs and TILs, respectively, was utilized as the clustering algorithm for data visualization and downstream analysis.

### 2.5 Multiplexed fluorescent immunohistochemistry

mIHC was performed on formalin-fixed paraffin-embedded (FFPE) tissues from 12 HCC patients (CIRB Reference No: 2016/2613). The tissues were stained with anti-human antibodies for CD8, PD-1 and CD38 (**Table S4**) using the OPAL™ 7-color IHC Kit (Perkin-Elmer) and DAPI. Images were acquired using Vectra 3.0 Pathology Imaging System Microscope (Perkin-Elmer) and analyzed using InForm v2.1 (Perkin-Elmer) and Imaris v9.1.0 (Bitplane).

### 2.6 Statistics

All statistical analyses were performed using GraphPad Prism (V9.0). Comparisons of cell frequencies between groups were done using the non-parametric one-way ANOVA Kruskal-Wallis test with Dunn’s posthoc multiple comparison test. The paired two-tailed Wilcoxon-matched T-test or unpaired two-sided T-test with Welch’s correction were applied for pairwise comparisons. Spearman’s Regression Analysis was used for correlation analysis.

## 3 Results

### 3.1 CD38 expression profile in PBMCs, NILs and TILs from HCC patients

To investigate CD38 expression in HCC, we used CyTOF to profile PBMCs, NILs and TILs obtained from 17 HCC patients (**Table S1**) with an antibody panel of 41 surface and intracellular immune markers (**Table S2**). In addition, to account for intratumoral heterogeneity (15), two to five tumor sectors (n=60) were collected from patients along with matched NILs (n = 17) and PBMCs (n = 17) samples.

**Figure 1:**
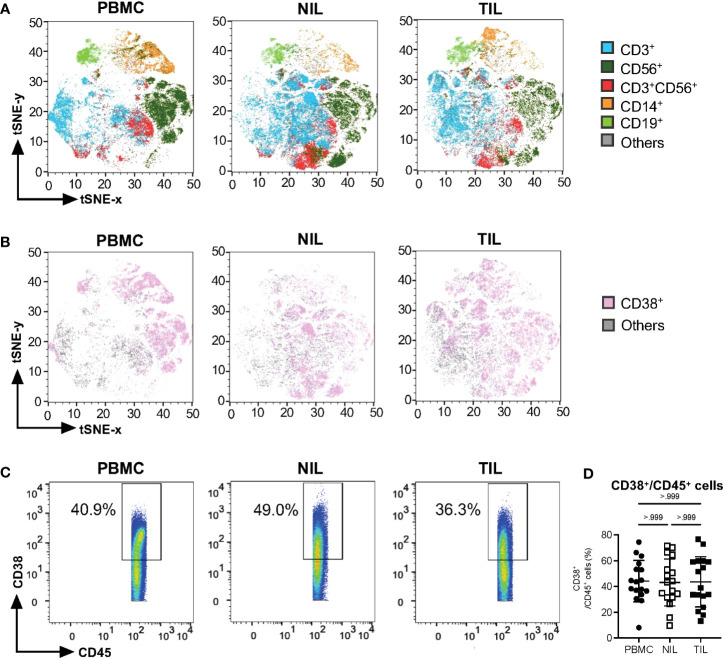
Characterization of CD38 expression profiles in PBMC, NIL and TIL of HCC. **(A)** tSNE plots showing five major immune lineages from PBMC, NIL and TIL. Each immune cluster is represented by one color. **(B)** tSNE plots demonstrating ubiquitous CD38 expression on five immune subsets in PBMC, NIL and TIL. **(C)** Representative manual gating of total CD38^+^CD45^+^ immune cells from the PBMC, NIL and TIL. **(D)** Percentage of CD38^+^ immune cells in PBMC, NIL and TIL. Data is represented as mean ± SD. Friedman one-way ANOVA test calculated by Dunn’s *post-hoc* multiple pairwise comparisons was performed. **(A, B, D)** PBMC, peripheral blood mononuclear cells (n=17); NIL, Non-tumor-infiltrating lymphocyte (n=17); TIL, tumor-infiltrating lymphocyte (n=60 from 2-5 tumor sectors per case).

Two-dimensional tSNE plots were generated from the CyTOF data, showing individual cells clustered based on the similarity in the expression of each immune marker (**Figure 1A**). The immune cells were clustered into five major immune lineages: CD3^+^ T cells, CD56^+^ Natural Killer (NK) cells, CD3^+^CD56^+^ NKT cells, CD14^+^ monocytes and CD19^+^ B cells (**Figure 1A**). tSNE plots based on CD38 expression show ubiquitous expression across major lineages from TILs, NILs and PBMCs (**Figure 1B**). There is no significant difference in the proportions of total immune cells expressing CD38 (CD38^+^CD45^+^) across PBMCs, NILs and TILs (**Figures 1C, D**). However, each compartment exhibited a distinct CD38^+^ immune profile (**Figures 1A, B**).

### 3.2 Elevated CD38 expression on CD3^+^ T cells in HCC TILs

**Figure 2:**
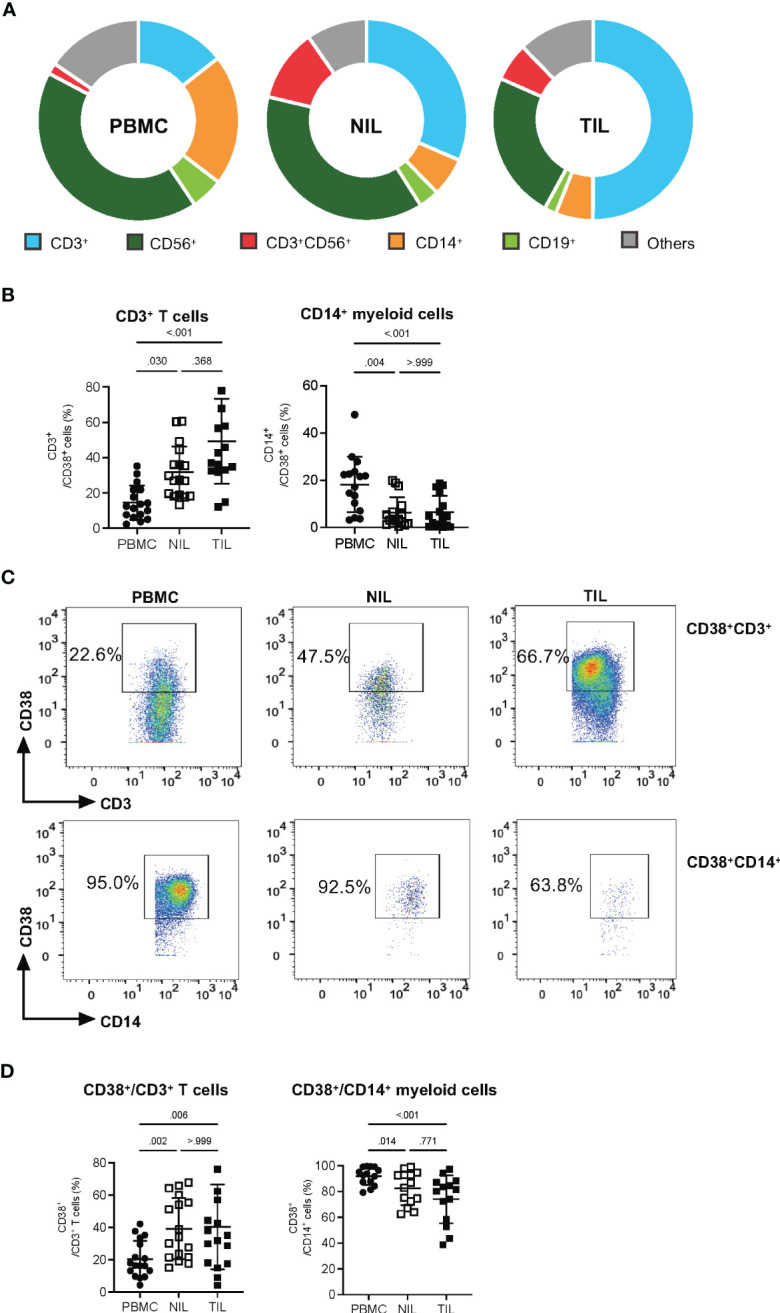
Elevated CD38 expression in CD3^+^ TILs. **(A)** CD38^+^ compositions from PBMC, NIL and TIL of HCC patients: CD3^+^ T cells, CD14^+^ myeloid cells, CD56^+^ Natural Killer (NK) cells, CD3^+^CD56^+^ NKT cells and CD19^+^ B cells. **(B)** Percentages of CD3^+^ T cells and CD14^+^ myeloid cells among the total CD38^+^ immune cells. **(C)** Representative manual gating strategy to identify the CD38-expressing CD3^+^ T cells and CD14^+^ myeloid cells from PBMC, NIL and TIL. **(D)** Percentages of CD38^+^ populations among the CD3^+^ T cells and CD14^+^ myeloid cells in PBMC, NIL and TIL. **(B, D)** Data are represented as mean ± SD. Friedman one-way ANOVA test calculated by Dunn’s *post-hoc* multiple pairwise comparisons was performed. p-value <0.05 is considered as significant. **(A, B, D)** PBMC, peripheral blood mononuclear cells (n=17); NIL, Non-tumor-infiltrating lymphocyte (n=17); TIL, tumor-infiltrating lymphocyte (n=60 from 2-5 tumor sectors per case).

Despite the lack of significant differences in the proportions of CD38^+^CD45^+^ across PBMCs, NILs or TILs, a distinct CD38 expression profile could be observed (**Figure 1B**). We hence examined the compositions of specific immune subsets among the CD38-expressing immune cells by manual gating (**Figure S2A**). First, we observed that among the CD38^+^ immune cells, the proportion of CD19^+^ B cells is higher in the PBMCs than in the NILs and TILs; while CD56^+^ NK cells are significantly lower in the TILs as compared to the PBMCs and NILs; and the proportion of CD3^+^CD56^+^ NKT cells is significantly higher in the NILs and TILs compared to the PBMCs (**Figures 2A**, **S2B**). Consistent with a previous report (14), CD3^+^ T cells represent the main immune population that expresses CD38, particularly in TILs compared to PBMCs (**Figure 2B**). The CD14^+^ myeloid cells, on the other hand, follow the opposite trend, with the lowest proportion among the CD38^+^ immune cells in TILs as compared to PBMCs (**Figure 2B**).

Conversely, we also examined CD38 expression on individual immune subsets across PBMCs, NILs and TILs (**Figures 2C**, **S3A**). Consistent with our results above, the proportions of CD38^+^ populations among the total CD3^+^ T cells are significantly higher in TILs (**Figure 2D**). In contrast, the proportion of CD38^+^CD14^+^ myeloid cells is lower in TILs as compared to PBMCs (**Figure 2D**). On the other hand, the proportion of CD38^+^CD19^+^ B cells is higher in PBMCs than in NILs and TILs, the proportion of CD38^+^ NKT cell population is significantly lower in the PBMCs than in the NILs and TILs, and the proportion of CD38^+^CD56^+^ NK cells showed no significant differences across PBMCs, NILs and TILs (**Figure S3B**).

Taken together, the enriched proportion of CD3^+^ T cells among the CD38-expressing TILs suggests that CD38 may play a significant role in T cell immunity within the TME of HCC.

### 3.3 Tumor-infiltrating CD8^+^ tissue-resident memory T cells (T_RM_) is the dominant immune subset expressing CD38 in the HCC TME

**Figure 3:**
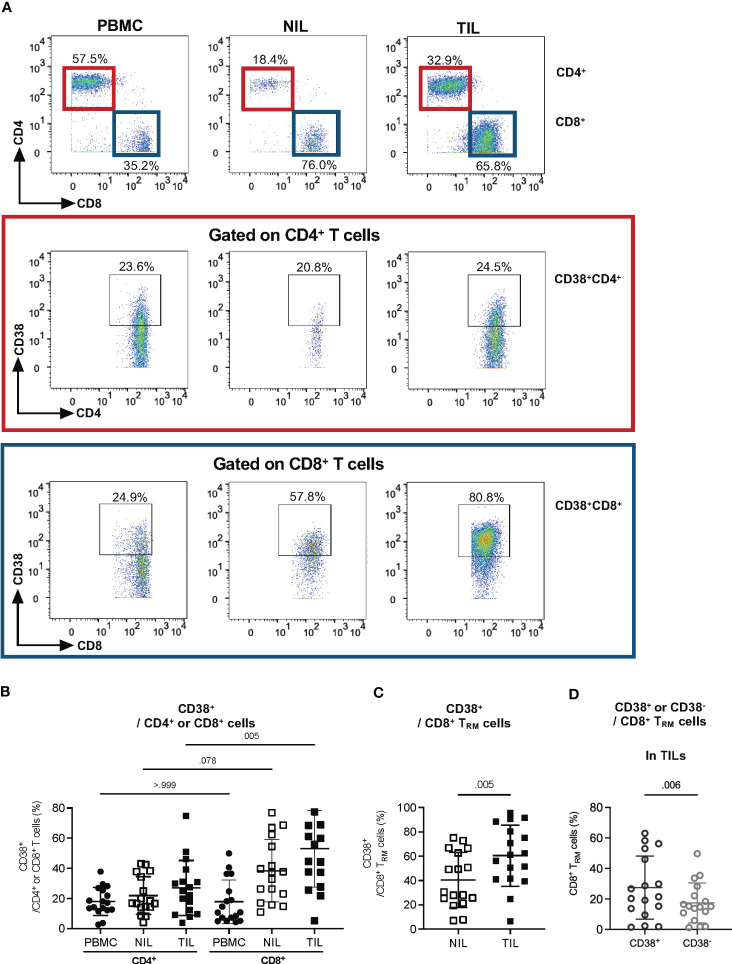
CD38 expression in tumor-infiltrating CD8^+^ Tissue-resident memory (T_RM_) cells. **(A)** Representative manual gating strategy for CD38-expressing CD3^+^ immune subsets, CD4^+^ and CD8^+^ T cells in PBMC, NIL and TIL. **(B)** Percentages of CD38-expressing CD4^+^ or CD8^+^ T cells in PBMC, NIL and TIL. Data is represented as mean ± SD. Friedman one-way ANOVA test calculated by Dunn’s *post-hoc* multiple pairwise comparisons was performed. p-value <0.05 is considered as significant. **(C)** The percentages of CD38^+^ T_RM_ among the CD8^+^ T cells in NILs and TILs. **(D)** The percentages of CD38^+^ and CD38^-^ among CD8^+^ T_RM_ in TILs. **(C, D)** Data are represented as mean ± SD. Two-tailed Wilcoxon-matched t-test was performed. p-value <0.05 is considered as significant. **(A–D)** PBMC, peripheral blood mononuclear cells (n=17); NIL, Non-tumor-infiltrating lymphocyte (n=17); TIL, tumor-infiltrating lymphocyte (n=60 from 2-5 tumor sectors per case).

Next, we sought to identify the specific CD3^+^ subset that expresses CD38. By gating on CD8^+^ or CD4^+^ T cells, a higher frequency of CD38^+^ can be observed on CD8^+^ T cells compared to that on CD4^+^ T cells, particularly on TILs, but not on the NILs and PBMCs (**Figures 3A, B**), suggesting that CD8^+^ T cells are the dominant CD38-expressing CD3^+^ T cells among the TILs in HCC tumors.

Since our previous study has shown that the HCC TME is enriched with CD8^+^ tissue-resident memory cells (T_RM_) that play an important role in tumor immunity (16), we evaluated CD38 expression on CD8^+^ T_RM_ (CD103^+^CD45RO^+^CD8^+^) in TILs and NILs (**Figure S4A**). Indeed, we observed a significantly higher frequency of CD38^+^ T_RM_ among the CD8^+^ T cells from TILs than that in the NILs (**Figure 3C**). Furthermore, within the TILs, the percentage of CD38^+^ T_RM_ is significantly higher than the CD38^-^ T_RM_ (**Figure 3D**), indicative of a specific role of CD38 on T_RM_ in the HCC TME.

### 3.4 Transcriptomic analysis of CD8^+^ T_RM_ cells from TILs revealed a T cell exhaustion signature associated with CD38

**Figure 4:**
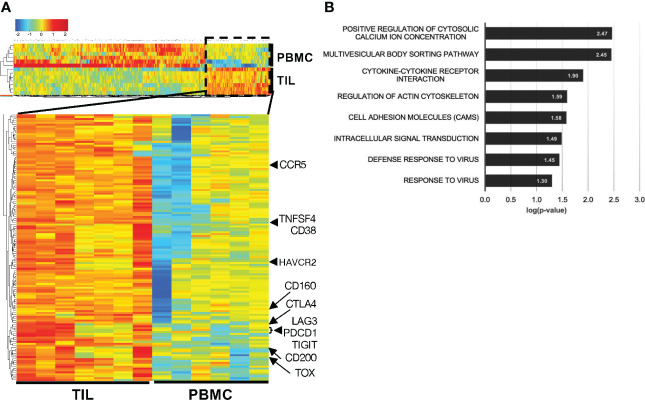
Higher *CD38* and exhaustion marker genes expression in the CD8^+^ T_RM_ from HCC TILs. **(A)** Differentially expressed genes (DEGs) between the T_RM_ extracted from the TILs (n=7) and circulating memory T cells extracted from the PBMCs (n=6). Cells were isolated from matched patient samples; one PBMC sample was omitted due to poor RNA quality. **(B)** DAVID Functional pathway analysis of the enriched DEGs from TIL-T_RM_ obtained from **(A)**. The genes of each pathway are listed in **Table S6**.

To further assess the role of CD38 in T_RM_ from HCC TILs, we sorted for CD8^+^CD103^+^CD45RO^+^ T_RM_ from TILs and circulating CD8^+^CD45RO^+^ memory CD8^+^ T cells from PBMCs (**Figure S1A**) and performed bulk RNA sequencing on the sorted populations. Analysis of the differentially expressed genes (DEGs) comparing these two immune populations showed significant enrichment of *CD38* in the CD8^+^ T_RM_ from TILs (**Figure 4A**, **Table S5**). In addition, we also observed elevated expression of the genes coding for T cell exhaustion markers, *PCDC1, CTLA4, HVCR2, LAG3* and *TIGIT* in CD8^+^ T_RM_ from TILs (**Figure 4A**, **Table S5**).

To further understand the genes associated with phenotypes of CD8^+^ T_RM_ from TILs, we interrogated their enriched DEGs by functional pathway analysis. Several significant pathways processes enriched in the tumor-infiltrating CD8^+^ T_RM_ include positive regulation of cytosolic calcium ion concentration (GO:0007204), multivesicular body sorting pathway (GO:0071985), cell adhesion molecules (CAM; hsa04514), intracellular signal transduction (GO:0035556), defense response to virus (GO:0051607) and response to virus (GO:0009615) (**Figure 4B**, **Table S6**). Intracellular calcium level is well known to affect T cell functions and activities (22) and is linked to T cell exhaustion (23). Several genes involved in the cell adhesion molecules pathway, such as cytotoxic T-lymphocyte-associated protein 4 (*CTLA4*) and programmed cell death protein 1 (*PDCD1*), are known to control T cell motility (24) and well-known T cell exhaustion markers as well (25).

Collectively, the targeted transcriptomics analyses support an association between CD38 and T cell exhaustion, specifically in T_RM_ from HCC TILs.

### 3.5 CD8^+^ T_RM_ cells in TILs co-express CD38 and multiple exhaustion makers

**Figure 5:**
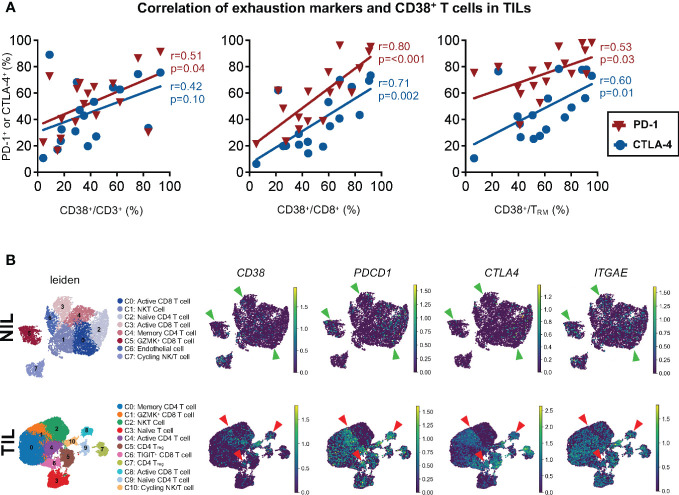
Association between CD38 and exhaustion markers on the tumor-infiltrating T cells. **(A)** Correlation between PD-1 or CTLA-4 with CD38 expression on CD3^+^ T cells (left), CD8^+^ T cells (middle) and CD8^+^ T_RM_ (right) from TILs. Spearman’s r and p-values are reported. p-value <0.05 denotes significant correlation. TIL, tumor-infiltrating lymphocyte (n=60 from 2-5 tumor sectors per case). **(B)** Single-cell RNA-seq data demonstrating the co-expression of *CD38*, *PDCD1* (PD-1), and *CTLA4* and *ITGAE* (CD103) in the NILs (top) and TILs (bottom). The green and red arrows indicate the CD8 T cell clusters in the NILs and TILs, respectively. The clusters were identified and annotated using the differentially expressed genes listed in **Tables S7** and **S8**.

Given the association of *CD38* with T cell exhaustion gene signature from the sorted CD8^+^ T_RM_ in TILs, we next sought to determine if CD38 correlates with exhaustion markers PD-1 and CTLA-4 in CD3 and CD8 TILs at the protein expression level using our CyTOF data. Within the HCC TILs, we observed the correlation of CD38 with PD-1 only on CD3^+^ T cells, but the correlation of CD38 expression with both PD-1 and CTLA-4 was observed on CD8^+^ T cells and CD8^+^ T_RM_ (**Figure 5A**). Importantly, a similar correlation between CD38 and T cell exhaustion markers was not observed in the NILs or PBMCs (**Figures S5A–C**), highlighting that the correlation is specific to CD8^+^ T cells or CD8^+^ T_RM_ in HCC TME. This data again marks CD38 as a co-exhaustion marker for CD8^+^ T cells in the TME of HCC.

To elucidate this further at the single-cell level, we next performed scRNA-seq on 14 NIL and TIL samples. We first sub-gated out the immune population of interest, T cells, from the NILs and TILs as previously described (21). Dimension reduction analysis was performed on a total of 7,628 and 26,441 T cells from NILs and TILs cells, identifying 8 and 11 clusters, respectively (**Figure 5B**). The clusters were identified and annotated according to the differentially expressed genes (DEGs) (**Tables S7, S8**). Among the TILs, co-expression of *CD38* with exhaustion markers, *PDCD1* and *CTLA4* (**Figure 5B**), as well as *ENTPD1* (CD39) (**Figure S6A**), was observed in CD8 T cell clusters 6 and 8, and the margin of cluster 1. However, this co-expression was not observed in NILs (**Figures 5B**, **S6A**). Furthermore, we also found that the expression of *ITGAE*, which encodes for tissue-resident marker CD103, overlapped with the expression of CD38 and the exhaustion markers in clusters 1, 6 and 8 in TILs but not in NILs (**Figure 5B**). These findings suggest that CD38 co-expression with other known exhaustion markers is a characteristic feature of CD8 T cell, specifically the T_RM_ (**Figure 5B**).

The scRNA-seq data demonstrated, at the single-cell level, the potential co-expression of CD38 and other exhaustion markers on the same CD8^+^ T cells or T_RM_ from HCC TME, further validating its role as a co-exhaustion marker in HCC TILs. In support of our data, the co-expression of CD38 with T cells exhaustion markers was also observed on dysfunctional or exhausted CD8^+^ T cells in other tumor models (26, 27).

### 3.6 *In-situ* co-expression of CD38 and exhaustion makers on CD8^+^ T cells

**Figure 6:**
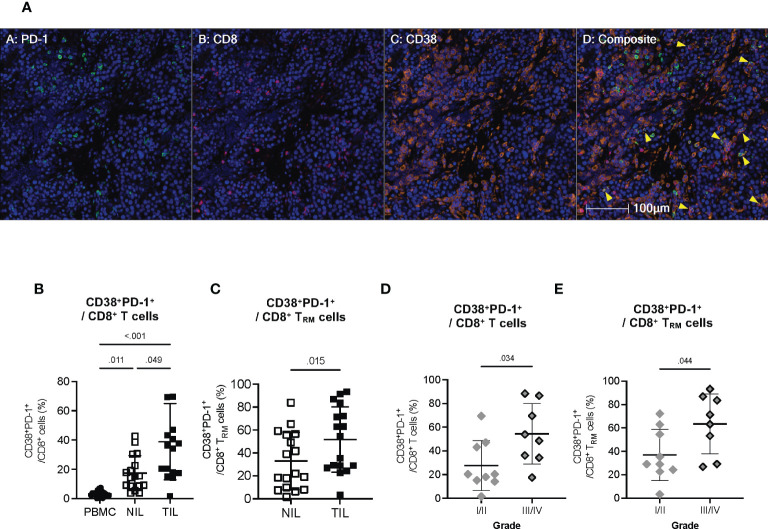
Co-expression of CD38 with exhaustion markers by the CD8^+^ T cells within TILs **(A)** Representative multiplex immunohistochemistry (mIHC) images showing co-expression of PD-1 (green), CD8 (red) and CD38 (orange) in the HCC TME. Yellow arrows designate co-expression of PD-1, CD8 and CD38. Cell nuclei are counterstained with DAPI (blue). Images are shown at 200x magnification. **(B)** Percentages of CD8^+^ T cells co-expressing CD38 and PD-1 in the PBMC, NIL and TIL. Friedman one-way ANOVA test calculated by Dunn’s post-hoc multiple pairwise comparisons was performed. **(C)** Proportion of CD8^+^ T_RM_ cells co-expressing CD38 and PD-1 in the NIL and TIL. Data is Two-tailed Wilcoxon-matched t-test was performed. **(D)** Percentages of tumor-infiltrating CD38^+^PD-1^+^CD8^+^ T cells in Edmonson-Steiner histopathological grade I or II versus III or IV. **(E)** Percentages of tumor-infiltrating CD38^+^PD-1^+^CD8^+^ T_RM_ cells in Edmonson-Steiner histopathological grade I or II versus III or IV. **(B–E)** Data represented as mean ± SD. p-value < 0.05 is considered as significant. PBMC: peripheral blood mononuclear cells (n=17); NIL: Non-tumor-infiltrating lymphocyte (n=17); TIL: tumor-infiltrating lymphocyte (n=60 from 2-5 tumor sectors per case). **(D, E)** Two-tailed Mann-Whitney t-test was performed. p-value < 0.05 denotes significant association. Clinical data from n=17 patients with HCC.

To visualize that CD38 is indeed expressed by the same exhausted CD8^+^ T cells, we performed multiplex immunohistochemistry (mIHC) on the formalin-fixed and paraffin-embedded (FFPE) tumor tissues from the HCC patients. We observed the colocalization of CD38, PD-1 and CD8 within HCC tumors (**Figure 6A**). To further validate that the same CD8^+^ T cells population does indeed co-express CD38 and PD-1, we manually gated them using our CyTOF data (**Figure S7A**). In line with our scRNA-seq data, we observed a higher frequency of CD8^+^ T cells co-expressing CD38 and PD-1 in the TILs compared to the NILs (**Figure 6B**). Specifically, a significant increase in the co-expression of CD38 and PD-1 was observed on CD8^+^ T_RM_ in TILs as compared to NILs (**Figure 6C**). Overall, our result suggests that the co-expression of CD38 with the immune checkpoint markers is specific to the CD8^+^ T_RM_ cells in the HCC TME.

Given the potential role of CD38 in T cell exhaustion, we next examined the association of the CD38^+^PD-1^+^CD8^+^ T cell subsets with the clinical parameters of our HCC patient cohort (**Table S1**). We found a significantly higher frequency of CD38^+^PD-1^+^CD8^+^ T cell and CD38^+^PD-1^+^CD8^+^ T_RM_ to be associated with HCC with higher histopathological grade III and IV (**Figures 6D, E**), indicating that CD38 as a T cell co-exhaustion marker is also linked to tumor aggressiveness.

Our study provided evidence that CD38 is closely associated with the T cell exhaustion signature, particularly on CD8^+^ T_RM_. This suggests that CD38, as another T exhaustion marker, could potentially be the next checkpoint molecule to be targeted for the immunotherapy of HCC.

## 4 Discussion

Our current study investigated the potential role of CD38 in HCC using an in-depth multi-dimensional immune profiling of the PBMCs, NILs and TILs obtained from patients with HCC. We demonstrated that CD38 is associated with T cell exhaustion in the HCC TME. Mainly, CD38 is co-expressed with PD-1 and CTLA-4 on tumor-infiltrating CD8^+^ T_RM_ cells, identifying CD38 as a potential immune checkpoint marker that could be harnessed for HCC immunotherapy.

The ubiquitous CD38 expression in different immune cell types shown in our current study is consistent with previous reports (7, 14). Notably in HCC, our current data demonstrated that PBMCs, NILs and TILs each showed a unique CD38 immune profile. Consistent with our earlier study (14), the current data demonstrated CD3^+^ T cells as the dominant CD38-expressing immune subset in TILs. Furthermore, among the CD3^+^ T cell subsets, we identified resident memory CD8^+^ T_RM_ cells (CD103^+^CD45RO^+^CD8^+^) as the specific T cell that expressed a high level of CD38 in the TME of HCC. In a study of non-small-cell lung cancer (NSCLC), CD38^+^CD8^+^T_RM_ was also observed within the TME (29) and was shown to be crucial for anti-tumor immunity in NSCLC (28). Here, we report that enrichment of CD38^+^CD8^+^T_RM_ in the HCC TME is associated with T cell exhaustion. We showed that CD38 is co-expressed with T cell checkpoint makers like PD-1 and CTLA-4 at both the RNA and protein levels, indicating the role of CD38 in CD8^+^ T_RM_ exhaustion. Indeed, the expression of CD38 in CD8^+^ T cells in TME has been associated with an immunosuppressive and dysfunctional phenotype that drives cancer progression (27). Moreover, CD38 could contribute to pro-tumoral TME by acting on other stromal cells and promoting hypoxia and angiogenesis (29, 30). Altogether, these underscore that inhibition of CD38 could potentially be a multi-faceted approach to reinvigorate the exhausted TME for immunotherapy.

Our pathway analysis of the enriched DEGs from isolated HCC T_RM_ shows enrichment of the pathway involved in positive regulation of the cytosolic calcium ion. CD38-mediated regulation of intracellular calcium signaling pathways in T cells has been previously reported (31). CD38 regulates nicotinamide adenine dinucleotide (NAD^+^) levels involved in intracellular calcium mobilization, resulting in a NAD-mediated suppression of T lymphocytes and poor anti-tumoral immunity in the TME (32), linking CD38 to T cell exhaustion or immune evasion mechanistically. Despite that, we acknowledge the limitation of our current data, which warrants a validation of the detailed mechanism of CD38-mediated T cell exhaustion in HCC. Furthermore, apart from the T cells, we also found other immune cells expressing CD38, including those of the myeloid lineage. A higher density of CD38^+^ tumor-associated macrophages (TAM) has been associated with improved prognosis (12) and better response to PD-1/PD-L1 therapy (13) in HCC. The association could be attributed to CD38^+^ TAMs exhibiting the pro-inflammatory M1 phenotype, contributing to anti-tumor immune activity (13). Henceforth, while we show CD38 expression in CD8^+^ T_RM_ may play a role in T cell exhaustion, the other tumor-infiltrating immune cells could also contribute to the overall tumor immunity of the HCC TME. Interestingly, this suggests a more intricate role of CD38 in the HCC TME.

Our study has limitations that should be considered. The small sample size and variability in the number of tumor sectors obtained from each patient in our cohort may limit the generalizability of our findings. However, we addressed this by validating our findings using an independent HCC cohort and confirming the co-expression of CD38 and PD1 in CD8^+^ T cells through mIHC. Previous studies support our current findings demonstrating that CD38 is an immunosuppressive molecule and a potential T cell exhaustion marker (26, 27, 33). For instance, expression of CD38 on T-cells is associated with poorer proliferation and reduced pro-inflammatory cytokine secretion (26, 27, 33). The inhibition of CD38 has been shown to reinvigorate the cytotoxic function and proliferative capacity of CD38^+^CD8^+^ T cells (26, 27). Notably, a recent study has highlighted an additional role of CD38 in sustaining the survival of exhausted CD8^+^ T cells (33). These indicate a potential role for CD38 in promoting T cell dysfunction and exhaustion.

Moreover, our data suggest a promising synergistic effect of targeting CD38 and PD-1 to potentially reinvigorate the exhausted T cells in the TME and overcome the resistance to anti-PD-1 therapy. A study in melanoma directly linked the induction of PD1^+^CD38^hi^CD8^+^ T cells to T cell dysfunctionality found in the TME of non-responding patients to PD-1 blockade (27). Interestingly, Chen and colleagues report that CD38 blockade could rescue lung cancer mouse models from acquired anti-PD-1 therapy resistance (26). Although a Phase I/II clinical trial on the concurrent blockade of CD38 and PD-1 in solid-tumor cancers reported poor efficacy without significant anti-tumor activity, post-therapeutic examination revealed about a 40% reduction in the frequency of CD38^+^ tumor infiltrates in the TME and the reinvigoration of peripheral T-cell activity (34). The outcome of the clinical trial may have been confounded by the limited availability of patient samples and differing treatment histories affecting the baseline CD38 expression of TILs (34). Also, the TME of HCC could differ from that of advanced NSCLC and metastatic castration-resistant prostate cancer. Considering the concurrent expression of CD38 and PD-1 on HCC CD8^+^ TILs and its association with the higher HCC histological grades in our HCC cohort, targeting CD38 in combination with PD-1 blockade could revive the cytotoxic function of exhausted CD8^+^ T_RM_ cells. The combination therapy may have a synergistic effect to improve the clinical outcomes of HCC patients. Therefore, our study gives a glimpse of a potential direction for developing novel immunotherapy for HCC. Further studies will be needed to investigate the effectiveness of CD38 blockade with PD-1 in treating HCC in preclinical and clinical settings.

Overall, we provide evidence of CD38 co-expression with other exhaustion markers on CD8^+^ T_RM_ cells in the HCC TME, underpinning the role of CD38 in T cell exhaustion. Our findings uncover CD38 as a promising immune checkpoint marker as well as a potential target for combination immunotherapy for the treatment of HCC.

## Data availability statement

The datasets presented in this study can be found in online repositories. The names of the repository/repositories and accession number(s) can be found below: NCBI under accession code GSE156625.

## Ethics statement

The studies involving human participants were reviewed and approved by SingHealth Central Institution Review Board (CIRB) and the National University Hospital (NUH) CIRB (CIRB Ref: 2016/2626, 2016/2613 and 2018/2112). The patients/participants provided their written informed consent to participate in this study.

## Author contributions

All authors listed have contributed to this study. MR and MO are involved in data curation and analysis, investigation and manuscript writing. JL, TL, WL, AC, BG and PC recruited patients and provided patient specimens and discussed the data. YL, PN, CL, MW and CC assisted in data acquisition and analysis and discussed the paper. JS and RD obtained and analyzed the scRNA seq data. JY prepared and provided tissue samples, data analysis and discussed the data. VC designed and led the study, performed the analysis and prepared the paper. All authors contributed to the article and approved the submitted version.
