# Supplementary material for: CD38 marks the exhausted CD8+ tissue-resident memory T cells in hepatocellular carcinoma
Source: Front Immunol. 2023 Jun 12;14:1182016. doi: 10.3389/fimmu.2023.1182016 (PMC10292929; doi:10.3389/fimmu.2023.1182016)
Supplement: Supplementary file 1 [file DataSheet_1.docx]

Supplementary Material

**
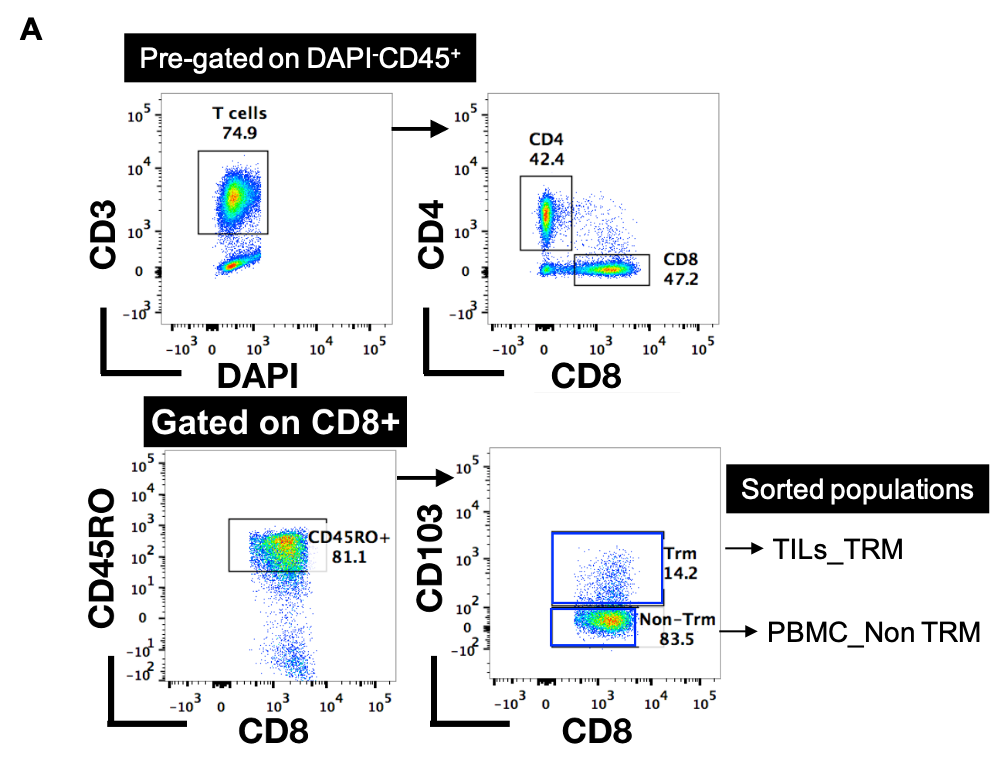
**

**Figure S1. Sorting Strategy for TILs and PBMCs. (A)** Representative plots for sorting of CD8^+^CD45RO^+^CD103^+^ T_RM_ from and CD8^+^CD45RO^+^CD103^-^ Non-T_RM_ (blue boxes) for subsequent bulk RNA-sequencing analysis. PBMC: peripheral blood mononuclear cells; TIL: tumor-infiltrating lymphocyte


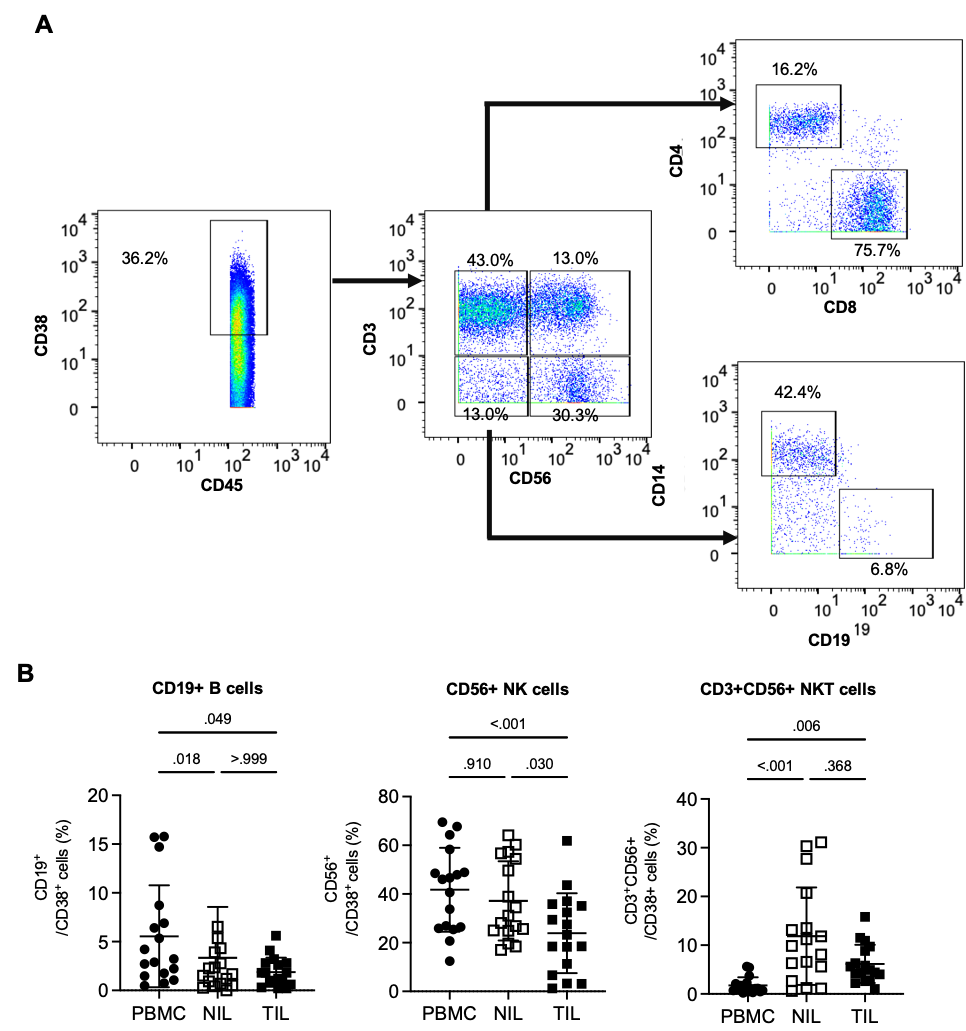


**Figure S2. CD38-expressing immune cells in PBMC, NIL & TIL of HCC. (A)** Representative gating strategy for identifying CD3^+^, CD56^+^, CD3^+^CD56^+^, CD14^+^, CD19^+^ cells among the CD38^+^ immune infiltrates in the TILs **(B)** Percentage of CD19^+^ B cells, CD56^+^ Natural Killer (NK) and CD3^+^CD56^+^ NKT cells among the total CD38^+^ immune cells. Friedman one-way ANOVA test calculated by Dunn’s post-hoc multiple pairwise comparisons was performed. p-values <0.05 denote statistical significance. PBMC: peripheral blood mononuclear cells (n=17); NIL: Non-tumor-infiltrating lymphocyte (n=17); TIL: tumor-infiltrating lymphocyte (n=60 from 2-5 tumor sectors per case).


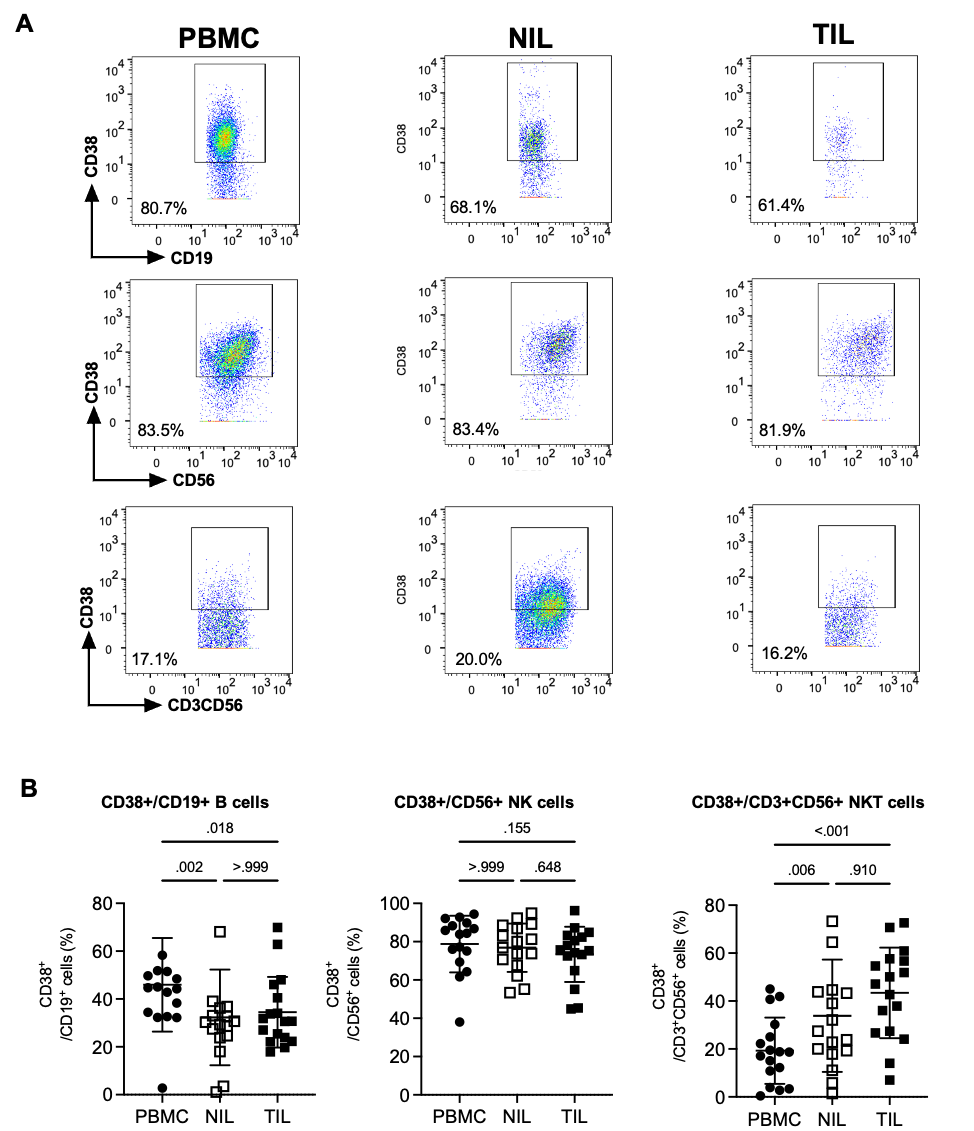


**Figure S3. CD38+ immune subsets analysis in PBMC, NIL & TIL of HCC. (A)** Representative manual gating strategy to identify the CD38-expressing CD19^+^ B cells, CD56_+_ Natural Killer (NK) and CD3_+_CD56^+^ NKT cells **(B)** Percentage of CD38^+^ cells among the CD19^+^ B cells, CD56^+^ NK and CD3^+^CD56^+^ NKT cells. Friedman one-way ANOVA test calculated by Dunn’s post-hoc multiple pairwise comparisons was performed. p-values <0.05 denote statistical significance. PBMC: peripheral blood mononuclear cells (n=17); NIL: Non-tumor-infiltrating lymphocyte (n=17); TIL: tumor-infiltrating lymphocyte (n=60 from 2-5 tumor sectors per case).


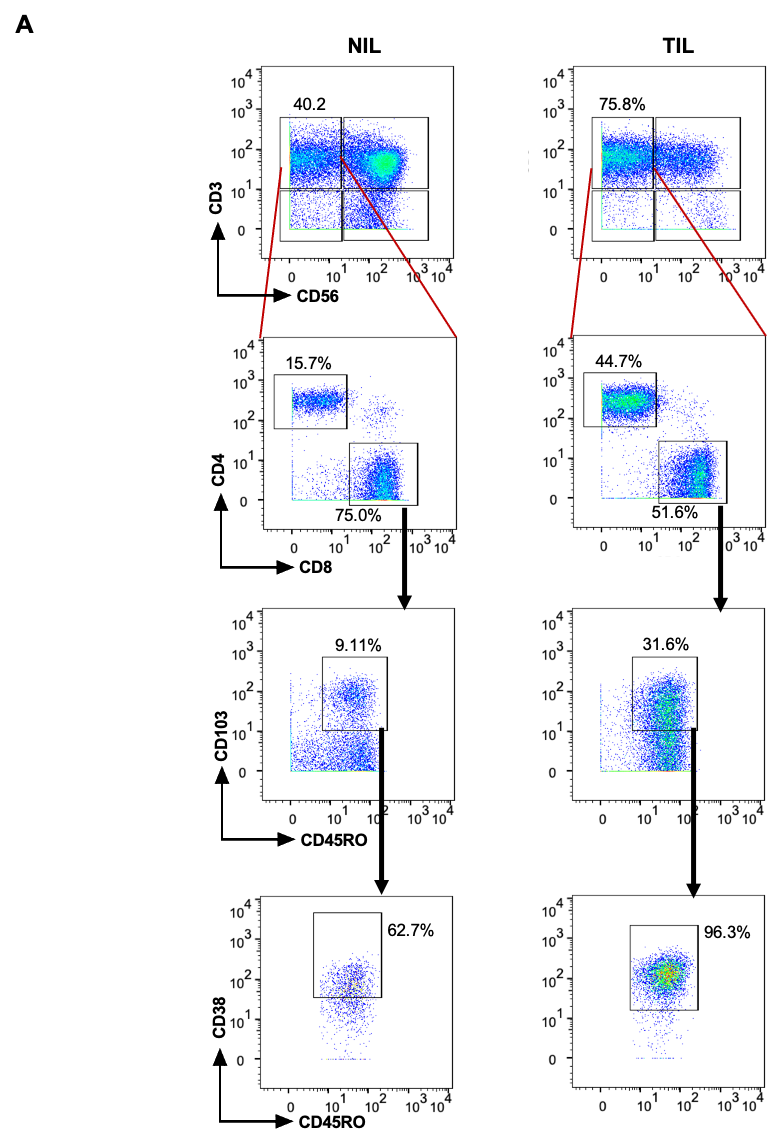


**Figure S4. Gating strategy for CD38+ T_RM_ (CD103^+^CD45RO^+^). (A)** Representative manual gating strategy to identify the CD38_+_ tissue-resident memory (T_RM_) cells expressing CD103 and CD45RO in NILs (left) and TILs (right). NIL: Non-tumor-infiltrating lymphocyte; TIL: tumor-infiltrating lymphocyte


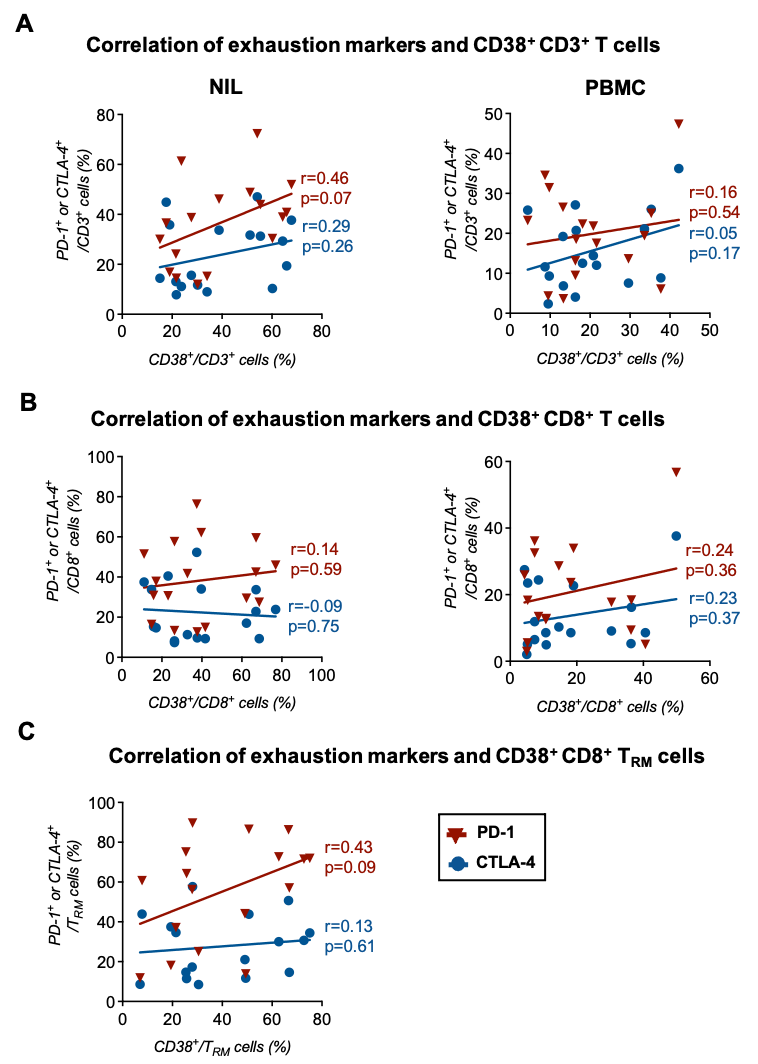


**Figure S5. Correlation between exhaustion markers and CD38 in CD3^+^, CD8^+^ and T_RM_ in the PBMCs and NILs.** Correlation between PD-1 or CTLA4 with CD38 expression in (**A**) CD3^+^ T cells from the NILs and PBMCs (**B**) CD8^+^ T cells from NILs and PBMCs; and (**C**) CD8^+^ T_RM_ from the NIL. Each dot represents a patient. r and p-values are reported. p-values > 0.05 are not significant. PBMC: peripheral blood mononuclear cells (n=17); NIL: Non-tumor-infiltrating lymphocyte (n=17)


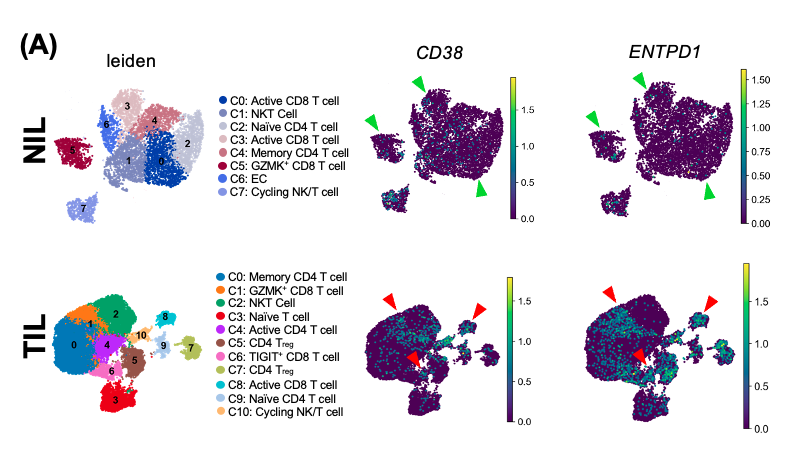


**Figure S6. Association between CD38 and CD39 in the CD8 clusters of NILs and TILs. (A)** Single-cell RNA-seq data showing the association of *CD38* with another T-cell exhaustion marker, *ENTPD1 (CD39)*, in the NILs (top) and TILs (bottom). The green and red arrows denote the CD8 T cell clusters in the NILs and TILs, respectively. The clusters were identified and annotated using the differentially expressed genes listed in Table S5 and S6.


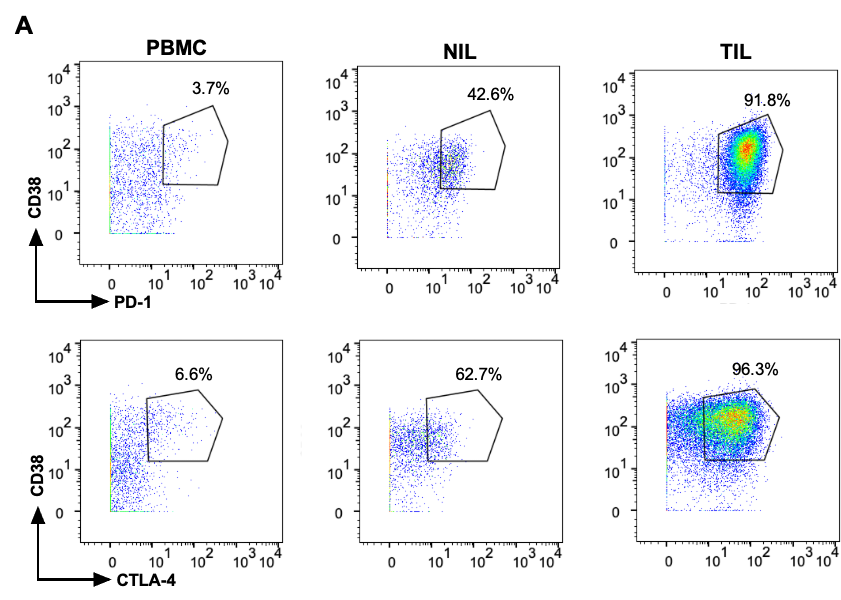


**Figure S7. Gating strategy for co-expression of CD38 with PD1 and CTLA4. (A)** Representative manual gating strategy to identify the coexpression of CD38_+_ with PD-1 or CTLA4 in CD8+ T cells in PBMCs, NILs, and TILs. PBMC: peripheral blood mononuclear cells (n=17); NIL: Non-tumor-infiltrating lymphocyte (n=17); TIL: tumor-infiltrating lymphocyte (n=60 from 2-5 tumor sectors per case).

**Table S1.** Clinical and demographic information of HCC patients (N=17)

| **Pat ID** | **Age** | **Gender** | **Race** | **Stage**  **(TNM)** | **Grade**  **(Edmondson)** | **Viral**  **status** | **Tumour size (cm)** | **No. of**  **sectors** | **Tumour**  **multiplicity** | **AFP level**  **(ng/ml)** | **MVI** |
| --- | --- | --- | --- | --- | --- | --- | --- | --- | --- | --- | --- |
| A008 | 70 | F | Malay | IB | II | NV | 3.8 | 2 | 1 | 248 | N |
| A009 | 47 | F | Chinese | II | III | NV | 7.5 | 4 | 1 | 2203 | Y |
| B015 | 63 | M | Chinese | IB | II | Hep B | 2.2 | 2 | 1 | 6.1 | N |
| B016 | 71 | M | Chinese | IB | II | Hep B | 4.7 | 4 | 1 | 8920 | N |
| B017 | 75 | M | Chinese | III | III | Hep B | 5.1 | 5 | 1 | 504 | Y |
| B018 | 46 | M | Chinese | III | III | Hep B | 4.6 | 4 | 1 | 657 | N |
| B019 | 63 | M | Chinese | II | II | Hep B | 5.7 | 5 | 2 | 62 | Y |
|  |  |  |  |  |  |  | 1.1 |  |  |  |  |
| C008 | 76 | M | Chinese | IIIA | II | Hep B | 14.0 | 4 | 1 | 13.7 | N |
| C010 | 55 | M | Indian | II | II | NV | 14.9 | 5 | 1 | 9.3 | N |
| C011 | 71 | M | Chinese | II | II | Hep B | 4.0 | 3 | 1 | 6.5 | N |
| C012 | 71 | M | Chinese | II | III | NV | 14.0 | 5 | 1 | 54458 | Y |
| C015 | 75 | M | Chinese | II | II | Hep B | 12.0 | 5 | 1 | 2.8 | Y |
| H156 | 66 | M | Chinese | III | III | Hep B | 2.3 x 2.1 | 2 | 1 | 4.3 | N |
| H194 | 72 | F | Other | I | III | NV | 6.9 | 1 | 1 | 27917 | N |
| H200 | 87 | M | Chinese | I | III | NV | 7.2 | 1 | 1 | 10.4 | N |
| H261 | 57 | M | Malay | II | III | Hep C | 2.2; 1 | 1 | 2 | 13.4 | N |
| H526 | 43 | M | Others | II | II | Hep B | 7.2 x 4.2 x 2.3 | 5 | 1 | 2985 | Y |

**Legend:**

Gender: M- Male; F-Female

Stage TNM: Version 8

Viral Status: Hep B- Hepatitis B; NV - non-viral-related HCC (patients with no detectable HBV surface/core antigen)

AFP: Alpha-fetoprotein

MVI: microvascular invasion

N- No; Y- Yes

**Table S2.** Antibodies used for Cytometry Time-of-Flight (CyTOF) staining

| **Isotopes** | **Antibodies** | **Clone** | **Vendor** |
| --- | --- | --- | --- |
| 89 | CD45 (Barcode 1) | HI30 | Fluidigm |
| 112/114 | CD14 | TüK4 | Lifetechnologies |
| 115 | CD45 (Barcode 2) | HI30 | Biolegend |
| 139 | HLA-DR | L243 | Biolegend |
| 141 | CD19 | HIB19 | Biolegend |
| 142 | CD45RO | UCHL1 | Biolegend |
| 143 | CD3 | UCHT1 | Biolegend |
| 144 | CD8 | SK1 | Biolegend |
| 145 | T-bet | 4B10 | Biolegend |
| 146 | TNFa | Mab11 | Biolegend |
| 147 | PD-1 | EH12.2H7 | Biolegend |
| 148 | CD4 | SK3 | Biolegend |
| 149 | VISTA | MAB71261 | R&D Systems |
| 150 | CD103 | B-Ly7 | Ebioscience |
| 151 | TIGIT | MBSA43 | Ebioscience |
| 152 | NKp46 | 9E2 | Biolegend |
| 153 | CD25 | 2A3 | BD bioscience |
| 154 | CD27 | O323 | Biolegend |
| 155 | CTLA-4 | BNI3 | BD bioscience |
| 156 | PD-L1 | 29E.2A3 | Biolegend |
| 157 | CD244 | C1.7 | Biolegend |
| 158 | IL-10 | JES3-9D7 | Biolegend |
| 159 | LAG-3 | 17B4 | Abcam |
| 160 | TIM-3 | F38-2E2 | Biolegend |
| 161 | CCR7 | G043H7 | Biolegend |
| 162 | CD56 | NCAM16.2 | BD bioscience |
| 163 | CXCR3 | G025H7 | Biolegend |
| 164 | GITR | 621 | Biolegend |
| 165 | FoxP3 | PCH101 | Ebioscience |
| 166 | Ki67 | 20Raj1 | Ebioscience |
| 167 | CD80 | 2D10 | Biolegend |
| 168 | IFN-y | B27 | Biolegend |
| 169 | IL-17A | BL168 | Biolegend |
| 170 | CCR6 | G034E3 | Biolegend |
| 171 | CD45RA | JS-83 | Ebioscience |
| 172 | CD45 (Barcode 3) | HI30 | Biolegend |
| 173 | GranzymeB | CLB-GB11 | Abcam |
| 174 | CD137 | 4B4-1 | Biolegend |
| 175 | CCR5 | T21/8 | Biolegend |
| 176 | CD69 | FN50 | Biolegend |
| 191/193 | Ir Intercalator | | Fluidigm |
| 209 | CD16 | 3G8 | Fluidigm |

**Table S3.** Antibodies used for Flow sorting

| **Antibodies** | **Fluorochrome** | **Clone** | **Vendor** |
| --- | --- | --- | --- |
| CD3 | APC | HIT3a | Biolegend |
| CD4 | BV605 | OKT4 | Biolegend |
| CD45 | APC-Cy7 | HI30 | Biolegend |
| CD45RO | AF700 | UCHL1 | Biolegend |
| CD8 | PerCP-Cy5.5 | RPA-T8 | Biolegend |
| CD103 | FITC | Ber-ACT8 | Biolegend |

**Table S4.** Antibodies used for multiplex immunohistochemistry (mIHC)

| **Antibodies** | **Clone** | **Vendor** |
| --- | --- | --- |
| CD8 | 4B11 | Leica Biosystems |
| PD-1 | NAT105 | Cell Marque |
| CD38 | SPC32 | Leica Biosystems |
| DAPI | - | Akoya Biosciences |

**Table S5.** Genes upregulated in the CD8^+^ tissue-resident memory T cells (T_RM_) from TILs vs CD8^+^ memory cells from the PBMCs

| **Ensembl IDs** | **Gene Name** | **logFC** | **PValue** | **FDR** |
| --- | --- | --- | --- | --- |
| ENSG00000114423 | CBLB | -1.0155 | 0.0144 | 0.4013 |
| ENSG00000277632 | CCL3 | -1.0207 | 0.0193 | 0.4452 |
| ENSG00000115738 | ID2 | -1.0407 | 0.0127 | 0.3779 |
| ENSG00000143669 | LYST | -1.0757 | 0.0122 | 0.3754 |
| ENSG00000077420 | APBB1IP | -1.0778 | 0.0225 | 0.4580 |
| ENSG00000100599 | RIN3 | -1.1367 | 0.0346 | 0.5338 |
| ENSG00000069974 | RAB27A | -1.1842 | 0.0212 | 0.4546 |
| ENSG00000100503 | NIN | -1.2163 | 0.0442 | 0.5819 |
| ENSG00000118503 | TNFAIP3 | -1.2272 | 0.0043 | 0.2405 |
| ENSG00000188389 | PDCD1 | -1.2845 | 0.0126 | 0.3779 |
| ENSG00000027075 | PRKCH | -1.3154 | 0.0018 | 0.1685 |
| ENSG00000103222 | ABCC1 | -1.3586 | 0.0319 | 0.5338 |
| ENSG00000112308 | C6orf62 | -1.3633 | 0.0265 | 0.4969 |
| ENSG00000135679 | MDM2 | -1.3689 | 0.0423 | 0.5668 |
| ENSG00000154451 | GBP5 | -1.3881 | 0.0471 | 0.6011 |
| ENSG00000091490 | SEL1L3 | -1.4158 | 0.0348 | 0.5338 |
| ENSG00000181847 | TIGIT | -1.4167 | 0.0123 | 0.3754 |
| ENSG00000091317 | CMTM6 | -1.4880 | 0.0067 | 0.2762 |
| ENSG00000091972 | CD200 | -1.4971 | 0.0048 | 0.2514 |
| ENSG00000143641 | GALNT2 | -1.5249 | 0.0422 | 0.5668 |
| ENSG00000170776 | AKAP13 | -1.5398 | 0.0002 | 0.0594 |
| ENSG00000109861 | CTSC | -1.5568 | 0.0023 | 0.1929 |
| ENSG00000100450 | GZMH | -1.5832 | 0.0297 | 0.5095 |
| ENSG00000053254 | FOXN3 | -1.6114 | 0.0360 | 0.5387 |
| ENSG00000140105 | WARS | -1.6492 | 0.0274 | 0.5040 |
| ENSG00000124406 | ATP8A1 | -1.6757 | 0.0331 | 0.5338 |
| ENSG00000118515 | SGK1 | -1.6793 | 0.0475 | 0.6046 |
| ENSG00000118922 | KLF12 | -1.6980 | 0.0095 | 0.3331 |
| ENSG00000134313 | KIDINS220 | -1.7061 | 0.0382 | 0.5489 |
| ENSG00000104043 | ATP8B4 | -1.7213 | 0.0025 | 0.1953 |
| ENSG00000177494 | ZBED2 | -1.7327 | 0.0004 | 0.0790 |
| ENSG00000104447 | TRPS1 | -1.7437 | 0.0158 | 0.4216 |
| ENSG00000213626 | LBH | -1.7604 | 0.0330 | 0.5338 |
| ENSG00000145016 | RUBCN | -1.7638 | 0.0367 | 0.5403 |
| ENSG00000132199 | ENOSF1 | -1.7759 | 0.0279 | 0.5040 |
| ENSG00000158636 | EMSY | -1.7927 | 0.0232 | 0.4640 |
| ENSG00000170802 | FOXN2 | -1.8194 | 0.0345 | 0.5338 |
| ENSG00000198846 | TOX | -1.8197 | 0.0195 | 0.4460 |
| ENSG00000140575 | IQGAP1 | -1.8203 | 0.0280 | 0.5040 |
| ENSG00000166780 | C16orf45 | -1.8437 | 0.0292 | 0.5074 |
| ENSG00000141337 | ARSG | -1.8518 | 0.0384 | 0.5507 |
| ENSG00000176890 | TYMS | -1.8566 | 0.0420 | 0.5654 |
| ENSG00000089775 | ZBTB25 | -1.8730 | 0.0391 | 0.5541 |
| ENSG00000141376 | BCAS3 | -1.8890 | 0.0363 | 0.5391 |
| ENSG00000074706 | IPCEF1 | -1.9069 | 0.0029 | 0.2073 |
| ENSG00000198382 | UVRAG | -1.9217 | 0.0335 | 0.5338 |
| ENSG00000164463 | CREBRF | -1.9278 | 0.0463 | 0.5945 |
| ENSG00000119778 | ATAD2B | -1.9426 | 0.0335 | 0.5338 |
| ENSG00000162636 | FAM102B | -1.9445 | 0.0424 | 0.5668 |
| ENSG00000008513 | ST3GAL1 | -1.9546 | 0.0182 | 0.4417 |
| ENSG00000095397 | WHRN | -1.9825 | 0.0164 | 0.4287 |
| ENSG00000105851 | PIK3CG | -2.0106 | 0.0207 | 0.4539 |
| ENSG00000151474 | FRMD4A | -2.0222 | 0.0285 | 0.5051 |
| ENSG00000113360 | DROSHA | -2.0353 | 0.0409 | 0.5598 |
| ENSG00000008869 | HEATR5B | -2.0530 | 0.0345 | 0.5338 |
| ENSG00000050730 | TNIP3 | -2.0590 | 0.0345 | 0.5338 |
| ENSG00000136861 | CDK5RAP2 | -2.0614 | 0.0341 | 0.5338 |
| ENSG00000112038 | OPRM1 | -2.0700 | 0.0024 | 0.1953 |
| ENSG00000100453 | GZMB | -2.1038 | 0.0006 | 0.1018 |
| ENSG00000121039 | RDH10 | -2.1123 | 0.0032 | 0.2118 |
| ENSG00000115232 | ITGA4 | -2.1307 | 0.0072 | 0.2868 |
| ENSG00000023171 | GRAMD1B | -2.1481 | 0.0039 | 0.2273 |
| ENSG00000162869 | PPP1R21 | -2.1525 | 0.0217 | 0.4558 |
| ENSG00000154127 | UBASH3B | -2.1645 | 0.0392 | 0.5541 |
| ENSG00000114999 | TTL | -2.1678 | 0.0112 | 0.3619 |
| ENSG00000115607 | IL18RAP | -2.1874 | 0.0289 | 0.5051 |
| ENSG00000134215 | VAV3 | -2.2005 | 0.0111 | 0.3614 |
| ENSG00000134996 | OSTF1 | -2.2028 | 0.0362 | 0.5391 |
| ENSG00000119685 | TTLL5 | -2.2203 | 0.0150 | 0.4146 |
| ENSG00000024862 | CCDC28A | -2.2765 | 0.0403 | 0.5589 |
| ENSG00000117281 | CD160 | -2.3125 | 0.0355 | 0.5378 |
| ENSG00000071553 | ATP6AP1 | -2.3267 | 0.0396 | 0.5546 |
| ENSG00000267680 | ZNF224 | -2.3296 | 0.0449 | 0.5856 |
| ENSG00000152217 | SETBP1 | -2.3431 | 0.0152 | 0.4146 |
| ENSG00000138767 | CNOT6L | -2.3478 | 0.0037 | 0.2253 |
| ENSG00000100714 | MTHFD1 | -2.3715 | 0.0422 | 0.5668 |
| ENSG00000163599 | CTLA4 | -2.3741 | 0.0017 | 0.1685 |
| ENSG00000173040 | EVC2 | -2.3868 | 0.0406 | 0.5598 |
| ENSG00000171848 | RRM2 | -2.3978 | 0.0169 | 0.4354 |
| ENSG00000006576 | PHTF2 | -2.4141 | 0.0191 | 0.4452 |
| ENSG00000138376 | BARD1 | -2.4271 | 0.0142 | 0.3980 |
| ENSG00000156475 | PPP2R2B | -2.4301 | 0.0393 | 0.5541 |
| ENSG00000197857 | ZNF44 | -2.4520 | 0.0278 | 0.5040 |
| ENSG00000071205 | ARHGAP10 | -2.4559 | 0.0117 | 0.3686 |
| ENSG00000087589 | CASS4 | -2.4577 | 0.0431 | 0.5692 |
| ENSG00000164187 | LMBRD2 | -2.4741 | 0.0365 | 0.5397 |
| ENSG00000106069 | CHN2 | -2.4780 | 0.0109 | 0.3571 |
| ENSG00000108798 | ABI3 | -2.4895 | 0.0222 | 0.4580 |
| ENSG00000196937 | FAM3C | -2.5069 | 0.0087 | 0.3126 |
| ENSG00000136631 | VPS45 | -2.5107 | 0.0419 | 0.5654 |
| ENSG00000198879 | SFMBT2 | -2.5161 | 0.0170 | 0.4354 |
| ENSG00000089692 | LAG3 | -2.5161 | 0.0069 | 0.2776 |
| ENSG00000148120 | C9orf3 | -2.5335 | 0.0111 | 0.3614 |
| ENSG00000138303 | ASCC1 | -2.5767 | 0.0021 | 0.1868 |
| ENSG00000131943 | C19orf12 | -2.5844 | 0.0324 | 0.5338 |
| ENSG00000162654 | GBP4 | -2.5980 | 0.0375 | 0.5449 |
| ENSG00000054983 | GALC | -2.6033 | 0.0202 | 0.4490 |
| ENSG00000120519 | SLC10A7 | -2.6701 | 0.0274 | 0.5040 |
| ENSG00000198825 | INPP5F | -2.6839 | 0.0213 | 0.4546 |
| ENSG00000198925 | ATG9A | -2.7558 | 0.0253 | 0.4838 |
| ENSG00000122335 | SERAC1 | -2.7722 | 0.0022 | 0.1896 |
| ENSG00000173905 | GOLIM4 | -2.7751 | 0.0226 | 0.4580 |
| ENSG00000160791 | CCR5 | -2.7793 | 0.0157 | 0.4216 |
| ENSG00000188313 | PLSCR1 | -2.7925 | 0.0321 | 0.5338 |
| ENSG00000128923 | FAM63B | -2.8153 | 0.0193 | 0.4452 |
| ENSG00000136111 | TBC1D4 | -2.8199 | 0.0025 | 0.1953 |
| ENSG00000111335 | OAS2 | -2.8248 | 0.0159 | 0.4216 |
| ENSG00000125726 | CD70 | -2.8590 | 0.0215 | 0.4558 |
| ENSG00000196189 | SEMA4A | -2.9681 | 0.0453 | 0.5874 |
| ENSG00000186187 | ZNRF1 | -2.9741 | 0.0102 | 0.3459 |
| ENSG00000186197 | EDARADD | -2.9923 | 0.0124 | 0.3777 |
| ENSG00000143970 | ASXL2 | -3.0053 | 0.0008 | 0.1195 |
| ENSG00000070540 | WIPI1 | -3.0219 | 0.0099 | 0.3376 |
| ENSG00000102547 | CAB39L | -3.0233 | 0.0336 | 0.5338 |
| ENSG00000102780 | DGKH | -3.0320 | 0.0094 | 0.3316 |
| ENSG00000137628 | DDX60 | -3.0741 | 0.0449 | 0.5856 |
| ENSG00000115020 | PIKFYVE | -3.1134 | 0.0116 | 0.3686 |
| ENSG00000186376 | ZNF75D | -3.1751 | 0.0030 | 0.2092 |
| ENSG00000136856 | SLC2A8 | -3.2109 | 0.0131 | 0.3824 |
| ENSG00000139193 | CD27 | -3.2376 | 0.0191 | 0.4452 |
| ENSG00000177888 | ZBTB41 | -3.2461 | 0.0325 | 0.5338 |
| ENSG00000105875 | WDR91 | -3.2660 | 0.0185 | 0.4452 |
| ENSG00000158290 | CUL4B | -3.3429 | 0.0142 | 0.3980 |
| ENSG00000163359 | COL6A3 | -3.6004 | 0.0163 | 0.4287 |
| ENSG00000181104 | F2R | -3.6179 | 0.0104 | 0.3478 |
| ENSG00000104490 | NCALD | -3.7008 | 0.0061 | 0.2687 |
| ENSG00000173064 | HECTD4 | -3.7954 | 0.0001 | 0.0524 |
| ENSG00000157601 | MX1 | -4.0489 | 0.0010 | 0.1246 |
| ENSG00000124191 | TOX2 | -4.2754 | 0.0044 | 0.2410 |
| ENSG00000136158 | SPRY2 | -4.4054 | 0.0350 | 0.5338 |
| ENSG00000149289 | ZC3H12C | -4.5103 | 0.0057 | 0.2687 |
| ENSG00000177409 | SAMD9L | -4.5195 | 0.0018 | 0.1685 |
| ENSG00000004468 | CD38 | -4.5475 | 0.0066 | 0.2758 |
| ENSG00000135077 | HAVCR2 | -4.6087 | 0.0004 | 0.0790 |
| ENSG00000121594 | CD80 | -4.6244 | 0.0140 | 0.3966 |
| ENSG00000136205 | TNS3 | -4.7494 | 0.0027 | 0.2013 |
| ENSG00000178445 | GLDC | -5.7279 | 0.0001 | 0.0323 |
| ENSG00000157483 | MYO1E | -6.2783 | 0.0005 | 0.0871 |
| ENSG00000117586 | TNFSF4 | -6.4660 | 0.0002 | 0.0555 |
| ENSG00000156234 | CXCL13 | -6.8107 | 0.0021 | 0.1868 |

**Table S6.** Differentially expressed genes and pathway analysis by Database for Annotation, Visualization and Integrated Discovery (DAVID)

| **Pathway name** | **Benjamini** | **p-value** | **Genes** |
| --- | --- | --- | --- |
| GO:0007204~  positive regulation of cytosolic calcium ion concentration | 0.471115 | 0.003393 | OPRM1, PIK3CG, CD38, CCR5, CXCL13, F2R |
| GO:0071985~  multivesicular body sorting pathway | 0.422506 | 0.00351 | UVRAG, RUBCN, RAB27A |
| hsa04060:  Cytokine-cytokine receptor interaction | 0.837394 | 0.012449 | CCL3, IL18RAP, TNFSF4, CCR5, CXCL13, CD70, CD27 |
| hsa04810:  Regulation of actin cytoskeleton | 0.846844 | 0.025548 | PIK3CG, VAV3, PIKFYVE, ITGA4, IQGAP1, F2R |
| hsa04514:  Cell adhesion molecules (CAMs) | 0.724817 | 0.026343 | TIGIT, CD80, CTLA4, ITGA4, PDCD1 |
| GO:0035556~  intracellular signal transduction | 0.906307 | 0.032316 | TNS3, SGK1, CD80, PIKFYVE, CHN2, PRKCH, AKAP13, DGKH |
| GO:0051607~  defense response to virus | 0.912639 | 0.035767 | PLSCR1, DDX60, LYST, OAS2, MX1 |
| GO:0009615~  response to virus | 0.950115 | 0.049905 | TNFSF4, DDX60, OAS2, MX1 |

**Table S7.** Differentially expressed genes for the annotation of the scRNA-seq clusters in the NILs (Figure 5B)

| NIL Clusters | | | | | | | |
| --- | --- | --- | --- | --- | --- | --- | --- |
| 0 | **1** | **2** | **3** | **4** | **5** | **6** | **7** |
| CCL4L2 | GNLY | RPS29 | XCL2 | C1orf56 | CRTAM | HBB | B3GNT7 |
| CCL4 | GZMB | RPS27 | XCL1 | RASGEF1B | CCL5 | HBA2 | TYROBP |
| CD69 | NKG7 | EEF1A1 | CCL5 | HNRNPH1 | RPS27 | IGKC | KLRF1 |
| GZMK | FGFBP2 | RPL39 | CCL4 | CDC42SE1 | CCL4 | IGLC2 | NKG7 |
| CD3D | GZMH | RPS12 | CCL4L2 | PPP1CB | RPS29 | APOA2 | FCER1G |
| CCL3L3 | PRF1 | RPS27A | KLRD1 | RP11-138A9.2 | CXCR4 | APOC3 | CMC1 |
| RP11-347P5.1 | KLRF1 | RPL21 | RP11-291B21.2 | CTNNB1 | MALAT1 | S100A8 | CD7 |
| GPR171 | SPON2 | RPL38 | CCL3 | B4GALT1 | GZMK | MGP | CLIC3 |
| RGS1 | KLRD1 | RPLP2 | RGS1 | WTAP | HLA-B | ALB | IFITM2 |
| IFNG | TYROBP | RPS28 | IFNG | C16orf54 | HLA-A | JCHAIN | CDC42SE1 |
| CD8B | FCGR3A | RPL37 | CD7 | PHKG1 | HLA-C | IGHG3 | KLRB1 |
| JAML | PTGDS | RPL37A | PARP8 | IL7R | RPL41 | IGLC3 | REL |
| THEMIS | S100B | RPL28 | CD8B | SET | TMSB4X | CXCL2 | KLRD1 |
| ATAT1 | PLAC8 | RPL13 | NR4A3 | MDM4 | SRGN | TM4SF1 | AREG |
| STAT1 | FCER1G | RPL9 | CD69 | EIF5A | TSC22D3 | ADIRF | GSTP1 |
| FKBP5 | CST7 | TPT1 | PIK3R1 | SERPINB9 | RPL23A | HLA-DRA | KLRC1 |
| ZNF780B | CMC1 | RPS21 | TYROBP | TRA2A | RGS1 | IL1B | C1orf56 |
| RASGRF2 | CD247 | RPS25 | ASXL2 | CDC42 | B2M | HP | CCL3 |
| ICOS | HOPX | RPL30 | TRGC2 | PRDM1 | EEF1A1 | IGHM | HNRNPH1 |
| RBM5-AS1 | AKR1C3 | RPS3A | AREG | TNRC6B | RPL39 | C10orf10 | CD160 |
| RGL2 | C12orf75 | RPL34 | MAP3K8 | ADAM19 | EIF1 | APOC1 | FAM177A1 |
| TRBC2 | C1orf21 | RPS16 | NR4A2 | LTB | HLA-E | TTR | B4GALT1 |
| NAP1L6 | LAIR2 | RPL23A | ID2 | PPP3CA | RPL13A | SPARCL1 | CD63 |
| RP11-140K17.3 | TRDC | RPL41 | REL | RPL36A | RPL36 | DNASE1L3 | VPS37B |
| CTD-2506J14.1 | CTSW | RPL36 | SYTL3 | PIM2 | ZFP36 | ADAMTS1 | SYTL3 |
| ZNF80 | HAVCR2 | MALAT1 | HCST | TTC39C | MT-ND2 | IGHG4 | XCL1 |
| HLA-DRB1 | IGKC | RPL11 | SDCBP | STK17B | RPLP2 | IFI27 | LAT2 |
| CCDC74A | PRSS23 | RPS23 | TTC39B | RP11-347P5.1 | RPL13 | APOA1 | CTSW |
| TPM2 | CYBA | RPL35A | STOM | RP11-51J9.5 | IL32 | GNLY | CCL4 |
| ARIH2OS | TTC38 | RPS15A | ZNF683 | CD40LG | RPS2 | IGHA1 | XCL2 |
| AIF1 | ADGRG1 | RPLP1 | TERF2IP | ZDHHC2 | RPS24 | IGHG1 | CD247 |
| MS4A7 | LGALS1 | RPL13A | CMC1 | APOBEC3C | RPS18 | IFITM3 | PLAC8 |
| SEC24D | PLEK | RPL32 | RP11-51J9.5 | RPS29 | DDX5 | TP53TG1 | IRF8 |
| CHPF2 | ZEB2 | RPS6 | ZFP36L1 | CCL20 | MT-CO1 | IGFBP7 | FOSL2 |
| METTL12 | THEMIS2 | RPL10 | TRIM23 | TSPYL1 | RPS28 | NNMT | PPP1CB |
| EDA2R | EFHD2 | RPS20 | ZNF331 | FOS | TUBA4A | C15orf48 | NR4A2 |
| IL1R2 | S1PR5 | RPS17 | N4BP2L1 | SETD5 | RPL27A | ITCH | TRDC |
| KRT7 | BPGM | RPL27A | CKLF | SLC4A10 | RPS6 | CALD1 | CDC42 |
| RP5-940J5.9 | TRGC1 | RPS4X | AC092580.4 | SVIP | RPL35A | S100A9 | LITAF |
| ERAP2 | IGHA1 | RPS14 | ITPRIPL2 | IL18R1 | RPL21 | LYZ | TMEM2 |
| SEPT8 | SYNGR1 | RPS8 | TMED3 | PLXNA3 | MT-ND3 | AKAP12 | GZMA |
| PARP8 | KIR2DL1 | RPL31 | PABPC1 | RP5-882C2.2 | CD69 | CXCL8 | IRF1 |
| TRAV36DV7 | SELPLG | RPS13 | NUDT14 | AC092580.4 | RPS25 | ELF3 | TRA2A |
| CTD-2371O3.2 | KIR2DL3 | RPL26 | CD160 | SRSF6 | RPS3 | IL33 | MCTP2 |
| HSPA4L | IGLC2 | RPS18 | NOL10 | ZC3H4 | PPDPF | ORM1 | PRF1 |
| CLEC4A | MTSS1 | RPL19 | IRF8 | GIGYF1 | RPL3 | FGB | IL2RB |
| TIFA | UBE2F | RPL18A | TMEM167B | ATP1B1 | H3F3B | IGHA2 | RHOC |
| GABBR1 | HSH2D | RPL12 | AC002331.1 | TNFAIP3 | RPS20 | CD9 | CD83 |
| AMZ2 | GK5 | RPL3 | ARL6IP5 | FAM76A | CCL4L2 | LTBR | YPEL5 |
| TP53INP1 | ITGAM | RPS3 | SPRY1 | RP5-1085F17.3 | RPL28 | S100A16 | EIF3G |
| MTFP1 | CX3CR1 | RPL7 | ZNRF2 | KLRB1 | ID2 | CRYAB | APMAP |
| HLA-DQA2 | CCL5 | RPS24 | ITGAD | PABPC1 | RPS19 | SPRY2 | CD38 |
| EML3 | RAMP1 | RPL14 | RP11-347P5.1 | SLC12A2 | RPS12 | AGXT | CHST12 |
| UBQLN4 | GTF3C1 | RPS2 | CCDC59 | LETM1 | RPL19 | RTKN2 | MAPK1 |
| GALR1 | LILRB1 | RPS7 | FCRL6 | APOL2 | RPL34 | INSR | APOBEC3C |
| HOXB2 | FGR | RPL7A | FOSB | JAML | RPL18A | HLA-DPA1 | TXK |
| RP11-755F10.1 | RAB29 | RPL18 | KIAA1147 | ADAM17 | RPL7 | GPX3 | TNFSF14 |
| RP11-400N9.1 | CD47 | RPL10A | DCTN6 | CAPZA1 | RPS15A | CCL2 | IFITM3 |
| EPB41L4A | TSPAN32 | RPS19 | GLA | AP1G1 | RPL30 | HIST1H2AC | SET |
| NSUN6 | KIR3DL2 | RPL22 | MEGF9 | CD69 | RPS4X | RAMP2 | BST2 |
| TRBV6-2 | MYO1F | RPL35 | RP13-580F15.2 | RIT1 | RPL37A | MIR181A2HG | SH2D1B |
| ERCC2 | PTPN12 | MT-ND3 | BABAM1 | TSPAN15 | RPL37 | HLA-DQB1 | CLDND1 |
| RP11-294N21.3 | CXCR2 | RPL5 | NPM2 | TTC39C-AS1 | ATP5E | EFNA1 | POLR2K |
| HLA-DQA1 | RASSF4 | RPL8 | ACSF2 | PIP4K2A | RPS14 | AMBP | AOAH |
| GBP5 | CEP78 | RPS9 | ZFR | RELT | CD8B | EGFL7 | C16orf54 |
| GPR155 | ABI3 | EIF1 | TOPBP1 | AMFR | CCNH | RNASE1 | ICAM1 |
| NFXL1 | CYP4F35P | RPL6 | SRRT | RBM6 | RPL15 | CPE | CST7 |
| HCRT | ANKRD20A4 | RPL27 | LDB2 | EGR1 | RPL10A | AZGP1 | PIK3R1 |
| KIZ | SLC15A4 | RPL24 | ZNF410 | RREB1 | MT-CO2 | FCER1A | CTNNB1 |
| LMOD1 | SH2D1B | UBA52 | SYTL2 | SNHG25 | FOS | RP11-1143G9.4 | MDM4 |
| RP11-134G8.7 | RP11-81H14.2 | RPS15 | SLC17A5 | CBWD3 | RPL9 | EMP1 | DUSP10 |
| NLGN2 | C1orf162 | RPLP0 | RAB37 | RP11-538P18.2 | ACTB | DEFB1 | TIPARP |
| PDK4 | COL6A2 | RPL15 | PPFIA1 | NOCT | RPL8 | FCN3 | BCO2 |
| FLT4 | GSAP | RPL29 | ACBD3 | RP11-147L13.12 | UBC | TIMP1 | DHRS3 |
| CCL20 | NME8 | RPS5 | MS4A1 | ICOS | RPL10 | RGS5 | DDX6 |
| MT1X | RAP1GAP2 | RPSA | ZSWIM4 | FCF1 | XCL2 | PLEKHH3 | GZMB |
| BCL2L11 | C5orf56 | MT-ND2 | RP11-47L3.1 | ANKRD40 | RPL32 | ABLIM3 | TMIGD2 |
| KIAA2012 | ITGA5 | RPS11 | MTHFR | ATF7IP2 | RPS7 | SPP1 | TWISTNB |
| MYLIP | AK5 | RPS10 | MIATNB | APOBEC3D | RPS27A | CLU | PPP3CA |
| SDC2 | HLA-DRB5 | B2M | MALSU1 | TAGLN | RPLP1 | TSPAN7 | DNAJB6 |
| TNF | CARD16 | FAU | PRMT9 | NUDT4 | CTSW | VTN | PIP4K2A |
| LINC00239 | CD300A | VIM | CHPT1 | ABRACL | CD3D | SLC13A5 | STARD3NL |
| SLC46A3 | TXK | FTH1 | HOPX | BAG1 | SARAF | C7 | SLA2 |
| ACTA2 | PCSK5 | EEF1B2 | UGGT1 | SPON1 | GZMA | SAA2 | GADD45B |
| RNGTT | SAMD3 | SARAF | PRNCR1 | GADD45G | KLF6 | NRP2 | SLC16A3 |
| FAM71F2 | EMP3 | HLA-A | IL11RA | AC013264.2 | RPL26 | SLC9A3R2 | SAR1A |
| SLC4A5 | SRPK2 | RPL23 | ZFP36L2 | DNAJC7 | HLA-DRB1 | SOCS3 | ITGAL |
| MAP3K7CL | CTBP2 | NACA | SAMSN1 | RQCD1 | MT-ND4 | LIMCH1 | TIGIT |
| FAM69A | DIP2A | RPL36A | FOS | ARHGEF26 | RPS3A | ADCY4 | IFRD1 |
| RP13-270P17.1 | STARD3NL | RPL4 | GOSR1 | OXNAD1 | RPL31 | KRT7 | ZNF331 |
| RPH3AL | CMTR2 | HSPA8 | APBB3 | TAF13 | RPL11 | CNN3 | SLC15A4 |
| RAD52 | TPST2 | HLA-B | MON2 | R3HDM2 | HCST | C8orf4 | MOB4 |
| CXCL8 | IGLC3 | IL7R | INADL | RP11-589C21.6 | ARHGDIB | RP11-6N17.6 | MAP3K8 |
| LINC01560 | SSBP3 | GNB2L1 | CYSLTR2 | KDM6B | RPL12 | ANG | LMNB1 |
| RP3-492J12.2 | HLA-DQA2 | GPR183 | YPEL5 | TNFRSF25 | RPS21 | CD36 | HSH2D |
| GNB5 | CXCR1 | NPM1 | ACSS1 | MTO1 | CD74 | TRIM59 | GYPC |
| SIRPG | LPCAT1 | UQCRB | NPEPL1 | TRAV4 | MT-CO3 | TNFAIP2 | PDCD4 |
| TRAV6 | PAFAH2 | CD44 | PRRT2 | RP11-452L6.1 | HNRNPA1 | ERG | IFITM1 |
| CTB-152G17.6 | SYNE1 | MT-ND1 | ST6GAL1 | ZNF652 | RPL27 | SAP130 | MIR181A1HG |
| ANO9 | FGL2 | LTB | CXorf38 | CHN1 | MT-ATP6 | FABP1 | SRGN |

**Table S8.** Differentially expressed genes for annotation of the scRNA-seq clusters in the TILs (Figure 5B)

| TIL Clusters | | | | | | | | | | |
| --- | --- | --- | --- | --- | --- | --- | --- | --- | --- | --- |
| 0 | **1** | **2** | **3** | **4** | **5** | **6** | **7** | **8** | **9** | **10** |
| IL7R | DONSON | NKG7 | CCR7 | HSPA1A | AC133644.2 | GEM | AC002331.1 | CXCL13 | MAPK13 | STMN1 |
| CD69 | JUNB | GNLY | RPL34 | HSPA1B | IL32 | ICA1 | IL32 | CCL5 | B2M | HMGB1 |
| KLRB1 | IGHG4 | GZMB | RPL32 | DNAJB1 | TIGIT | RGS1 | S100A4 | ITM2A | ACTB | HMGB2 |
| TXNIP | CRTAM | GZMH | RPS14 | HSPA6 | RGS1 | JUNB | CARD16 | RGS2 | HLA-A | TUBA1B |
| CD40LG | CCL5 | FGFBP2 | RPS27 | HSP90AA1 | CARD16 | TIGIT | B2M | RGS1 | HLA-C | HMGN2 |
| RPL34 | CREM | KLRD1 | RPLP2 | ZFAND2A | CD27 | TNFRSF9 | HLA-A | NKG7 | TMSB4X | ACTB |
| GPR171 | IGHG3 | TYROBP | RPL3 | HSPE1 | LAIR2 | CD27 | CTSC | CD8B | IL32 | TUBB |
| CCR6 | IGKC | CST7 | RPL13A | HSPH1 | HLA-A | PMCH | BATF | LYST | CFL1 | H2AFZ |
| LTB | ITM2B | PRF1 | RPS12 | DNAJA1 | BATF | LYST | TIGIT | FABP5 | EEF1A1 | GAPDH |
| TSC22D3 | RPS2 | CCL5 | RPS18 | HSPD1 | CTLA4 | ITM2A | S100A6 | CD27 | TMSB10 | PFN1 |
| CD52 | GZMK | TRDC | RPL11 | DNAJB4 | TRAC | ARID5B | ACTB | HLA-DRA | RPS15A | PTMA |
| GPR183 | CD8A | KLRF1 | EEF1A1 | HSP90AB1 | CTSC | PMAIP1 | TRAC | CD8A | MT-CO1 | PPIA |
| TRAT1 | MCL1 | FCGR3A | RPS3 | IFNG | TNFRSF4 | DUSP4 | TNFRSF18 | CD74 | RPS14 | KIAA0101 |
| RPS27 | RGS1 | PTGDS | RPL21 | HSPA8 | SAT1 | SRGN | MYL6 | HLA-DRB1 | TRAC | CFL1 |
| RPL41 | CST7 | CCL4 | RPS25 | CACYBP | OAZ1 | CTLA4 | TMSB10 | HLA-DPB1 | MALAT1 | HIST1H4C |
| AC013264.2 | HLA-DRB5 | CTSW | RPS27A | DNAJA4 | TNFRSF9 | ZNF331 | ATP5E | IGKC | EEF1D | TOP2A |
| XIST | SRSF2 | PLAC8 | RPS6 | NR4A1 | ARID5B | BTG1 | AC133644.2 | HLA-DPA1 | PTMA | CALM2 |
| AIM1 | ITM2A | FCER1G | RPS4X | PPP1R15A | ICOS | TBC1D4 | GAPDH | SRGN | RPSA | H3F3A |
| LST1 | FTH1 | HSP90AA1 | RPL10 | FOS | GAPDH | DONSON | SAMSN1 | TNFRSF9 | RPL15 | NUCKS1 |
| FKBP5 | GLTSCR2 | C1orf21 | RPL23A | UBB | B2M | IGKC | LAIR2 | CST7 | RPLP2 | COX8A |
| RP11-51J9.5 | BTG1 | PLEK | RPL39 | BAG3 | LINC00152 | UBC | TNFRSF4 | JUNB | RPS15 | HNRNPA2B1 |
| S100A4 | RGCC | ZEB2 | RPL36 | APOA2 | CD3D | CD8A | ENO1 | PARK7 | RPL13A | CKS1B |
| FKBP11 | CD8B | CD247 | RPL27A | ALB | UBC | PHLDA1 | CD7 | ZNF331 | RPS29 | NUSAP1 |
| RPL36 | YPEL5 | TRGC2 | RPL41 | HSPB1 | DUSP4 | CARD16 | SAT1 | TIGIT | LY6E | RAN |
| RP11-18H21.1 | RPS4Y1 | SPON2 | RPS15A | TNFSF14 | ACTB | ITM2B | LTB | HLA-DMA | RPL39 | TPI1 |
| RPS4X | XCL2 | APOA2 | RPL7 | APOC3 | ATP5E | HLA-A | CD3D | HLA-DQA1 | RPL37 | ACTG1 |
| TNFAIP3 | SRSF7 | TRGC1 | RPS28 | RGS2 | ARPC1B | MAGEH1 | ICOS | PCAT29 | HLA-E | MYL6 |
| RBMS1 | TUBA4A | CCL3 | RPS23 | SERPINH1 | TBC1D4 | TERF2IP | CD27 | OAZ1 | ATP5G2 | SERF2 |
| TTC39C-AS1 | CLEC2B | S100B | RPS29 | MRPL18 | PMAIP1 | RGS2 | GLRX | CD7 | CD2 | PSME2 |
| RPL18A | LYST | HSPA1A | RPL10A | DOK2 | CD74 | IGHG4 | IL2RG | KLRD1 | RPL3 | CALM3 |
| FOS | XCL1 | ALB | RPS8 | BTG2 | TNFRSF18 | NR4A2 | RGS1 | IGLC2 | GNB2L1 | ATP5L |
| ZBTB16 | IER2 | APMAP | RPL31 | FTL | FOXP3 | ENTPD1 | LGALS1 | HLA-DRB5 | RPS24 | SNRPB |
| RPS28 | HLA-DPB1 | LITAF | RPL35A | DUSP1 | GBP2 | FABP5 | CTLA4 | APOC3 | CD3E | CLIC1 |
| IFI44L | ACVR2B | GZMM | RPL28 | FOSB | CD2 | MT-ND2 | TMSB4X | AHI1 | RPS3 | UBE2C |
| CTD-3252C9.4 | CXCR4 | CHST12 | RPL30 | EGR1 | AC017002.1 | HLA-DRB5 | HLA-C | GAPDH | CORO1A | SUMO2 |
| RPL31 | FAM118A | CYBA | RPL18A | APOC1 | SOD1 | CREM | RPS27L | MS4A6A | FAU | ARPC2 |
| LINC00239 | SRGN | CMC1 | RPL9 | ORM1 | IL2RA | BATF | ARID5B | CCL4 | MYL6 | SLC25A5 |
| RIPK2 | NR4A2 | BPGM | IL7R | JUN | CXCR6 | CD200 | TBC1D4 | DUSP4 | MT-ATP6 | PTTG1 |
| ANKRD28 | JUN | RP11-81H14.2 | RPS13 | APOA1 | PKM | RPS2 | PMAIP1 | RAB27A | RPL19 | CENPF |
| RPS19 | ATP8A1 | PRSS23 | RPL26 | ID2 | CD7 | NR3C1 | GBP2 | ALOX5AP | RPL10A | CHCHD2 |
| CXCR4 | SERP1 | STARD3NL | RPL19 | IL32 | MYL6 | LAYN | ARPC1B | GEM | HLA-B | TMSB4X |
| CTSH | HMGN1 | DSTN | RPS15 | UBC | DNPH1 | IGHG3 | LINC00152 | PTPRC | RPS28 | SRP14 |
| FLT3LG | UQCRB | B3GNT7 | RPL38 | SLC5A3 | S100A4 | NMB | NAMPT | APOA2 | RPS13 | ARPC5 |
| TTC39C | GZMA | IGHG4 | RPL14 | AHSA1 | NGFRAP1 | OAZ1 | SOD1 | SH2D1A | RPL28 | HNRNPA3 |
| KLF6 | HLA-DRA | ADGRG1 | RPS19 | CD69 | ICA1 | TNFRSF18 | UBC | APOC1 | PSMB9 | CARHSP1 |
| ODF2L | DUSP4 | PYHIN1 | RPS3A | TXNIP | GLRX | RGCC | PKM | PDCD1 | RPL6 | COX6C |
| MGAT4A | IGLC3 | TTC38 | RPL35 | GADD45B | TRBC2 | WHSC1L1 | IL2RA | SNAP47 | CD3D | MT2A |
| CAPG | TERF2IP | TTR | RPS20 | PRMT9 | NAMPT | SNX9 | CFL1 | LIMS1 | RPS16 | PSMB9 |
| DPP4 | ZNF331 | KIR2DL3 | RPL18 | NUDC | FKBP1A | BTG3 | RPS26 | NR3C1 | ATP5E | PPP1CA |
| RPL35 | HLA-DPA1 | HSPH1 | RPS7 | ATF3 | SRP14 | LIMS1 | DUSP4 | MT-ND4 | HNRNPA1 | RHOA |
| GZMK | LEPROTL1 | PTPRE | RPS17 | APOH | MIR4435-2HG | LAIR2 | CORO1B | HLA-DQB1 | RPS4X | PSMA4 |
| PARP8 | VIM | GSTP1 | RPL13 | CCL4L2 | C9orf16 | FAM3C | PHLDA1 | H2AFZ | MT-CO3 | ATP5E |
| RUNX2 | TNFRSF9 | S1PR5 | RPS16 | CTSW | H3F3A | ICOS | DNPH1 | SRRT | RPS8 | CORO1A |
| RPS29 | UBE2S | HSPE1 | RPS9 | LGALS1 | UCP2 | CYCS | CD2 | XCL2 | ARPC1B | DEK |
| RPS18 | FAM46C | MATK | RPL8 | MALAT1 | PFN1 | NGFRAP1 | MALAT1 | CREM | RAC2 | CD74 |
| RPLP2 | FABP5 | SLC15A4 | RPLP1 | AMBP | HLA-B | SRSF2 | SERF2 | IFNG | S100A6 | HNRNPA1 |
| NLRP3 | STK17A | SYNGR1 | RPL5 | CHORDC1 | HLA-C | MS4A6A | FOXP3 | CTLA4 | RPL11 | TMSB10 |
| AQP3 | WHSC1L1 | KIR3DL2 | RPL12 | NEU1 | PARK7 | RHOH | FTL | GSTP1 | RPS19 | BLOC1S1 |
| RPL13 | EIF4A3 | FCRL6 | RPS5 | LPAR6 | RTKN2 | ID1 | UBE2B | TRGC2 | MT-ND4 | UBB |
| TPD52 | ZEB2 | UAP1 | RPL15 | MXD1 | RPS26 | MT-ND3 | CKLF | RNF19A | RPL23A | DBI |
| LEF1 | HLA-DQA1 | AOAH | TPT1 | GIMAP4 | RHBDD2 | LINC01480 | HLA-B | HOPX | RPS27 | HN1 |
| RPL12 | PNRC1 | ABI3 | RPL27 | NABP1 | IL2RG | STAT3 | UGP2 | PTTG1 | RPS11 | SEPT7 |
| RP11-347P5.1 | MAP1LC3B | DOK2 | RPL37 | CTD-3252C9.4 | PTPRC | CLEC2D | H3F3A | ZEB2 | RPL32 | H2AFV |
| FOSB | CCL4 | CD300A | RPL29 | HSD17B7 | CORO1B | CD8B | NGFRAP1 | MT-ATP6 | SH3BGRL3 | UQCRQ |
| IFI44 | HLA-DRB1 | AKNA | RPL37A | CLK1 | CREM | RNF19A | CLIC1 | HLA-A | RPL8 | LDHA |
| ZFP36L2 | MZT2B | APOBEC3G | FAU | GZMA | CALM3 | GLTSCR2 | LAYN | NR4A2 | RPL21 | ARL6IP1 |
| AC016831.7 | KPNA2 | FGR | RPSA | RBP4 | ENO1 | GPR18 | ARHGDIB | CMC1 | MYL12A | ARPC1B |
| RBL2 | RPL13 | MCTP2 | RPL6 | XIST | UBE2B | GADD45A | PFN1 | HCST | RPS6 | ANP32E |
| RPL32 | YBX1 | GTF3C1 | RPS21 | HP | SIRPG | PTMA | CXCR6 | APOA1 | H3F3B | ATPIF1 |
| RPS25 | NSMCE3 | GZMA | RPLP0 | SIT 1.00 | LTB | RTKN2 | TRBC2 | CRTAM | RPS20 | BIRC5 |
| SLC4A10 | RHOH | DNAJB6 | RPL36A | TTR | RPS27L | MT-ATP6 | ARL6IP5 | CYCS | ARPC2 | COX6A1 |
| RPL10A | PTMA | THEMIS2 | GNB2L1 | NUPR1 | HINT1 | SLA | RPS19 | HMGN1 | S100A4 | ERH |
| TRABD2A | IGLC2 | PRMT2 | RPL7A | ID3 | GBP5 | PCAT29 | SPOCK2 | SIRPG | OST4 | PSMB8 |
| RPL11 | SLC2A3 | SH2D1B | RPL4 | TLE4 | METTL8 | SAT1 | SUMO2 | ATP5E | GAPDH | ANP32B |
| MAL | PIK3R1 | LAT2 | UBA52 | TRA2B | SUB1 | TNFRSF1B | ALDOA | GZMA | EVL | RAC2 |
| ARHGAP15 | KRT10 | APOC3 | ANXA1 | MYL12A | GPX1 | NSMCE3 | COX8A | TERF2IP | RPS17 | PSMA7 |
| RP11-138A9.2 | UQCRFS1 | HSPD1 | PABPC1 | ANKRD37 | RHOH | IGFLR1 | ACTG1 | SRSF7 | RPL37A | CDK1 |
| RPL28 | RPS10 | APOC1 | RPL24 | SEPP1 | YWHAB | SOD1 | FKBP1A | FYB | RPLP1 | OAZ1 |
| CELF2 | PDIA3 | MAPK1 | RPS24 | ORM2 | ACP5 | SRSF7 | UBL5 | SRRM2 | SERF2 | SRSF3 |
| ANXA1 | CYCS | GK5 | RPL22 | PTPN7 | SUMO2 | SRRM2 | UCP2 | JAML | RPS27A | PA2G4 |
| RPLP0 | GUK1 | IFITM3 | RPS10 | HOPX | ATPIF1 | TGIF1 | RPL41 | FTL | RPL18A | SIVA1 |
| RP1-313I6.12 | GADD45GIP1 | ADRB2 | GPR183 | DNAJB6 | MAGEH1 | GK | COX7A2 | CD84 | RPL10 | SH3BGRL3 |
| DYNLT1 | MZT2A | AKR1C3 | MT-ND2 | APOE | HLA-DRB1 | H2AFZ | PARK7 | LIMD2 | RPL12 | MAD2L1 |
| ERN 1.00 | OASL | IFITM2 | TMSB10 | FKBP4 | CLEC2D | C20orf24 | OAZ1 | COTL1 | CD37 | DUT |
| RPLP1 | BCL11B | CX3CR1 | MT-CO3 | CMB9-22P13.1 | HLA-DMA | LAPTM4A | SUB1 | LSP1 | RPL34 | ATP5J2 |
| RORC | RNF19A | C12orf75 | EIF1 | CSF1 | UQCR11 | ID3 | BTG3 | UBC | RPL26 | POMP |
| TC2N | HLA-DQA2 | SAMD3 | SARAF | GBP3 | SAMSN1 | GLCCI1 | GPX1 | SRSF2 | ARPC3 | SUB1 |
| IFNGR1 | C20orf24 | PATL2 | ZFP36 | IER3 | MAF | JAK1 | VAMP8 | PSMB9 | CLIC1 | SNRPG |
| RASGRP2 | HERPUD2 | CD160 | CXCR4 | STIP1 | ARL6IP1 | STX11 | RPL8 | TRAC | ACTG1 | PSME1 |
| IL4I1 | INSIG1 | LYAR | EEF1B2 | TRAF3IP3 | ENTPD1 | PNRC1 | SH3BGRL3 | TNFRSF1B | GPSM3 | RPLP1 |
| RPL3 | FAM177A1 | SH2D2A | MALAT1 | FABP1 | COX8A | SHFM1 | BIRC3 | TOX | HNRNPA2B1 | ANAPC11 |
| IL17A | PITHD1 | HSP90AB1 | EEF2 | AHSG | PIM2 | IKZF2 | PRDX1 | NMB | RPL35A | SMC2 |
| C10orf54 | RNMT | IGHG3 | RPL23 | CCL3L3 | LSP1 | AHI1 | STK17B | GLTSCR2 | RPL36 | TMEM258 |
| OXNAD1 | GADD45G | RAB29 | MT-CO2 | TNF | NDUFA4 | PTP4A1 | MIR4435-2HG | CCL4L2 | RPL18 | RPA3 |
| AC058791.1 | PPP2CA | CD63 | EIF3E | CKS2 | BTG3 | TNFRSF4 | LSP1 | HLA-DQA2 | LAMTOR4 | ATP5G3 |
| IL26 | DDX3Y | TGFBR3 | TSC22D3 | RGS16 | CCND2 | UBE2S | ATP5G2 | TRBC2 | RPL27A | NDUFB2 |
| PPP2R5C | RANBP2 | RP5-1171I10.5 | TMSB4X | TAF7 | ICAM3 | CAV1 | CD3E | YBX1 | HCST | LSM 5.00 |
| S100A6 | GPR18 | EFHD2 | MT-CO1 | SOD1 | HNRNPK | GBP2 | CYTIP | ATPIF1 | RPS12 | PSMB3 |
| TMEM63A | EIF1AY | HP | PTMA | ALDOB | PGAM1 | HMGN1 | UQCR11 | ALB | RPS9 | ARHGDIB |
| PIK3IP1 | GGA2 | MYO1F | PFDN5 | SGK1 | LAYN | TSHZ2 | TNFRSF9 | SHFM1 | PSME2 | TK1 |
